# Severe impairment of male reproductive organ development in a low SMN expressing mouse model of spinal muscular atrophy

**DOI:** 10.1038/srep20193

**Published:** 2016-02-02

**Authors:** Eric W. Ottesen, Matthew D. Howell, Natalia N. Singh, Joonbae Seo, Elizabeth M. Whitley, Ravindra N. Singh

**Affiliations:** 1 Department of Biomedical Sciences, Iowa State University, Ames, Iowa 50011, USA; 2 Department of Veterinary Pathology, Iowa State University, Ames, Iowa 50011, USA

## Abstract

Spinal muscular atrophy (SMA) is caused by low levels of survival motor neuron (SMN), a multifunctional protein essential for higher eukaryotes. While SMN is one of the most scrutinized proteins associated with neurodegeneration, its gender-specific role in vertebrates remains unknown. We utilized a mild SMA model (C/C model) to examine the impact of low SMN on growth and development of mammalian sex organs. We show impaired testis development, degenerated seminiferous tubules, reduced sperm count and low fertility in C/C males, but no overt sex organ phenotype in C/C females. Underscoring an increased requirement for SMN expression, wild type testis showed extremely high levels of SMN protein compared to other tissues. Our results revealed severe perturbations in pathways critical to C/C male reproductive organ development and function, including steroid biosynthesis, apoptosis, and spermatogenesis. Consistent with enhanced apoptosis in seminiferous tubules of C/C testes, we recorded a drastic increase in cells with DNA fragmentation. SMN was expressed at high levels in adult C/C testis due to an adult-specific splicing switch, but could not compensate for low levels during early testicular development. Our findings uncover novel hallmarks of SMA disease progression and link *SMN* to general male infertility.

Spinal muscular atrophy (SMA) is a leading genetic cause of infant mortality^1^ and results from deletion and/or mutation of *survival motor neuron 1* (*SMN1*), a gene that codes for SMN protein^1^. A nearly identical gene, *SMN2*, cannot compensate for the loss of *SMN1* due to predominant skipping of exon 7, producing SMNΔ7, a truncated protein that is partially functional and highly unstable^2^. The severity of SMA correlates with the level of SMN, a multifunctional protein implicated in regulation of small nuclear heteronuclear ribonucleoprotein (snRNP) biogenesis, transcription, translation, stress granule formation, signal transduction and axonal transport of mRNA (references in Seo *et al*., 2013^3^). Motor neurons are particularly sensitive to the loss of SMN, although reduced SMN independently affects non-neuronal tissues, including muscle^4^, heart^5^, lungs, and intestine^6^. SMN is required for male germ cell maintenance in *Drosophila*^7^, however, no parallel can be drawn with mammalian spermatogenesis, which occurs within the specialized microenvironment of seminiferous tubules.

Multiple mouse models recapitulating various aspects of SMA have been generated^8^. The Taiwanese and Δ7 mice, the best characterized and most widely utilized models, exhibit severe phenotypes, including early postnatal lethality, impaired maturation of neuromuscular junctions and overall deficient motor function^1^,^8^. However, due to their short lifespan, these models are not appropriate for examining the role of SMN in male reproductive organ development. The testis is unique in producing higher SMN levels due to predominant expression of full-length *SMN2* transcripts compared to all other adult tissues and organs of a mild mouse model of SMA^9^,^10^. Alternative splicing of several genes are switched during testicular development^11^. However, it is not known if *SMN2* exon 7 undergoes a similar switch.

The recently reported *Smn*^*C/C*^ (C/C) model expresses a reduced amount of SMN (~25–50% of WT) and displays a mild SMA-like phenotype, including peripheral necrosis, autonomic nervous system dysfunction and allodynia^12^. Here we employ the C/C mouse to examine the role of SMN in reproductive organ development. We observed reduced testis size and impaired spermatogenesis in C/C mice, despite high SMN expression in testis. We show severe perturbations of the testicular transcriptome in young adult C/C mice, suggesting massive reprogramming of transcription and posttranscriptional regulation. Our results uncover a surprising shift in splicing regulation of various *SMN2* exons during the first wave of spermatogenesis. Our findings implicate for the first time a role for SMN in mammalian reproductive organ development and further elucidate the complex physiological role of this essential protein.

## Results

### C/C males exhibit small testes with impaired spermatogenesis and reduced fertility

**Figure 1:**
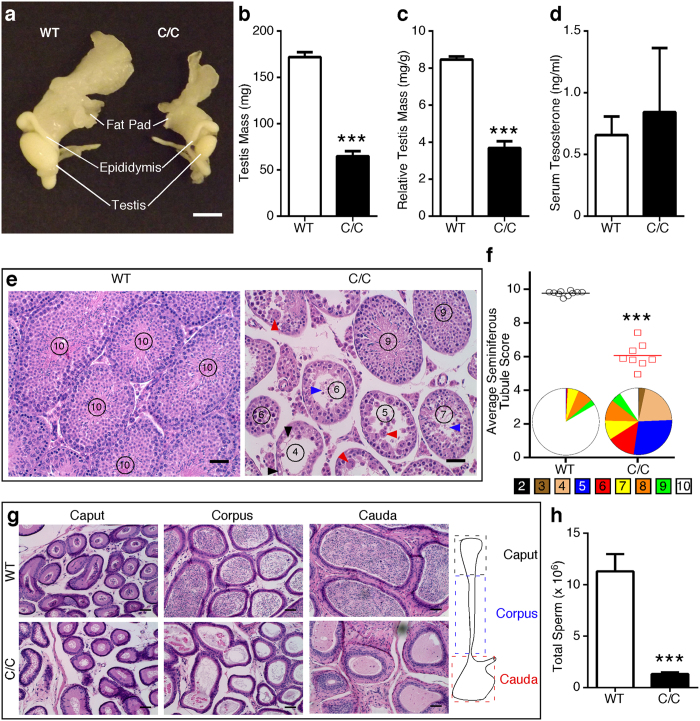
Male reproductive organs and functions are abnormal in P42 C/C mice. (**a**) Representative photograph of WT (left) and C/C (right) male reproductive organs, including testis, epididymis and epididymal fat pad. Scale bar represents 5 mm. (**b**) Gross testis mass. (**c**) Relative testis mass was determined by dividing gross testis mass by total body weight (n = 10 WT and 6 C/C mice). (**d**) Serum testosterone level determined by ELISA (n = 5 mice per genotype). (**e**) Representative H&E stained testis cross-sections. Circled numbers represent score of each tubule based upon a 10 point system that assesses morphology^13^. Damage and degeneration, including vacuolization (black arrowheads), multinucleated bodies (red arrowheads) and sloughed tissue (blue arrowheads) are indicated. Scale bars represent 50 μm. (**f**) Average seminiferous tubule scores for WT and C/C testes. Each point represents a single mouse (n = 10 WT and 6 C/C mice). Pie charts below data points represent the distribution of all seminiferous tubule scores for WT and C/C males. The legend underneath indicates the color for each score. (**g**) Representative H&E stained cross sections of epididymal regions. The cartoon to the right indicates the location of each region. Scale bars represent 50 μm. (**h**) Total epididymal sperm count from P60 males (n = 8 WT and 6 C/C mice). (Statistical significance: ***p < 0.001).

To determine the effect of low SMN on reproductive organ development, we examined wild type (WT) and C/C mice at postnatal day 42 (P42). At this age, mice have completed the first wave of spermatogenesis. Notably, we observed substantially smaller testes in C/C males even after correcting for body weight (Fig.1a–c). Testosterone is a crucial hormone regulating testis development, however, we observed no significant difference in serum testosterone between WT and C/C mice (Fig. 1d). Histological analyses of C/C testes showed heterogeneity of seminiferous tubules, with evidence of degeneration, including vacuolization, multinucleated bodies and sloughed cells (Fig. 1e, right panel). We assessed the health of seminiferous tubules using an established scale^13^. The average score for C/C testes indicated an overall reduction in post-meiotic cells and disrupted spermatogenesis (Fig. 1f). Histology of C/C epididymis showed a lack of or reduced number of spermatozoa in tubules of all regions (Fig. 1g). Consistently, we observed a ~10-fold reduction in epididymal sperm count in P60 C/C males compared to WT males (Fig. 1h), indicating that spermatogenesis was impaired, but not globally arrested.

**Figure 2:**
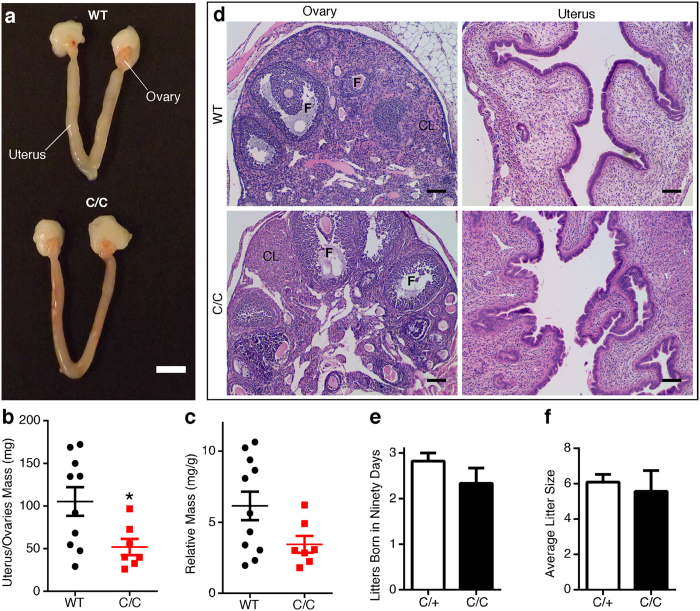
P42 female C/C reproductive organs exhibit minimal abnormalities. (**a**) Representative photograph of WT (top) and C/C (bottom) uterus and ovaries. Scale bar represents 5 mm. (**b**) Gross uterus and ovaries mass. (**c**) Relative uterus and ovaries mass was determined by dividing gross uterus and ovaries mass by total body weight (n = 10 WT and 7 C/C mice). (**d**) Representative H&E stained cross-sections of ovary and the endometrium. Follicle (F) and corpus luteum (CL) are indicated on ovary micrographs. Scale bars represent 100 μm. (**e–f**) Fertility of heterozygous (n = 11) and C/C (n = 3) female mice. Females were paired in monogamous breeding cages with either a heterozygote or WT male. The average number of litters born over ninety days (**e**) and the average litter size for heterozygote or C/C mothers (**f**) were recorded. (Statistical significance *p < 0.05).

Unlike testes, C/C female reproductive organs appeared normal, although variable in size (Fig. 2a). Gross uterus/ovaries mass was reduced for C/C females (Fig. 2b), but not after correction by total body weight (Fig. 2c). Beyond the gross weight difference, there were no overt histological changes in C/C uterus and ovaries (Fig. 2d). Ovaries from females of both genotypes exhibited follicles with oocytes in various stages, and the corpus luteum and uterus appeared normal (Fig. 2d).

In order to evaluate the fertility of C/C mice, we set up breeding cages with different combinations of WT, C/C, and heterozygous (C/+) mice (Supplementary Table 1) and followed them for ninety days. WT males all sired multiple litters of pups (Supplementary Table 1). In contrast, only two of the eight C/C males sired a litter: one male paired with a WT female and another paired with a C/C female (Supplementary Table 1). Both sired only one litter and neither female appeared pregnant again after giving birth. The fact that two C/C males were able to sire at least one litter despite very low sperm count suggests no debilitating defect in sperm motility and/or fertilization. In contrast, C/C females were fertile; over a 90 day period, there was no significant difference in number of litters born (Fig. 2e) or litter size (Fig. 2f) compared to heterozygote females (Supplementary Table 1).

### High levels of SMN are expressed in C/C testis

**Figure 3:**
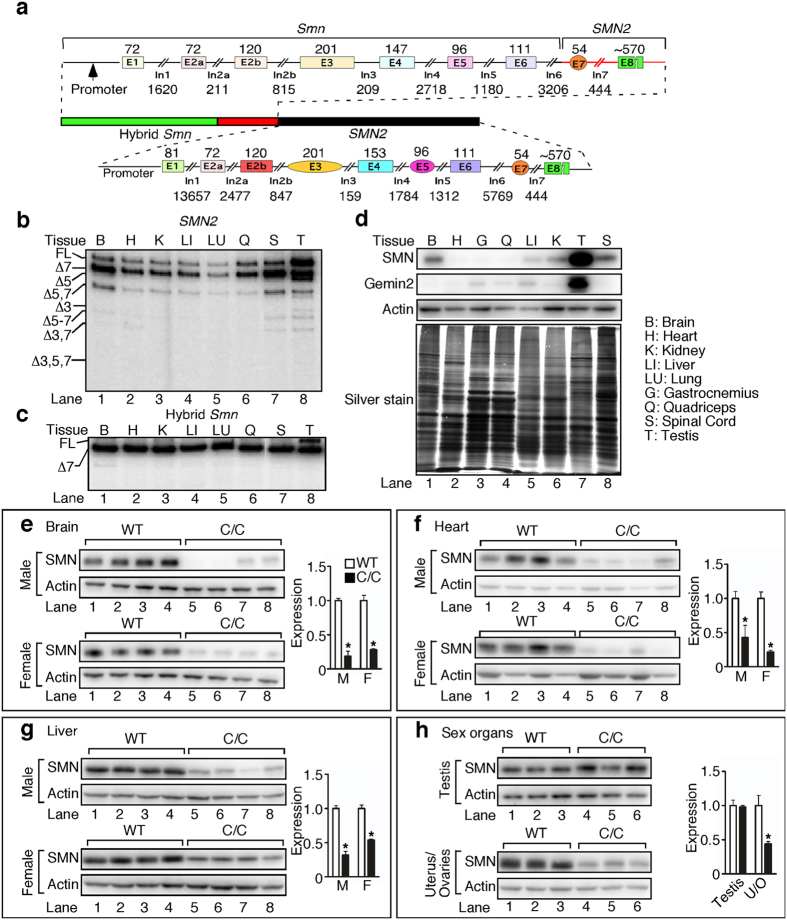
High levels of SMN are expressed in C/C testis. (**a**) Diagram of the *Smn*^*C*^ locus. Endogenous mouse *Smn* is interrupted 2,195 bp into intron 6 and replaced with the equivalent human *SMN2* sequence through the 3′-UTR, followed by 42 kb human *SMN2* genomic sequence. Exon lengths appear above colored boxes and intron lengths appear below lines. This transgene results in two genes, hybrid *Smn* and WT *SMN2*. MESDA^14^ was used to determine all splice variants for *SMN2* (**b**) and *Smn* hybrid (**c**) in various tissues. Band identities are given to the left of the lanes, where Δ indicates the lack of specific exon(s). Tissue abbreviations are indicated in panel (**d**). Additional abbreviations: E, exon; In, intron; UTR, untranslated region; FL, full-length. (**d**) Western blots of SMN, Gemin2 and Actin (top three panels) expression in adult WT tissue, and accompanying silver stain to confirm approximately equal loading. Samples were prepared by dissecting tissues from 3 WT males and immediately grinding them together in liquid nitrogen. 5 μg of protein was loaded for silver staining and 7.5 μg was loaded for western blotting. Tissue abbreviations appear to the right. (**e–h**) Representative SMN and Actin western blots from male (M) and female (F) brain (**e**), heart (**f**), liver (**g**), and testis and uterus/ovaries (U/O) (**h**). For (**e–h**), n = 3 or 4 mice per genotype. (Statistical significance: *p < 0.05).

The recently developed multi-exon-skipping detection assay (MESDA) determines the relative abundance of all *SMN* splice isoforms in a single reaction^14^. We employed MESDA on various tissues of C/C mice. Consistent with previous reports^9^,^10^, testis emerged as the only tissue to have full-length *SMN2* transcript as the major product (Fig. 3b), and the only tissue to express appreciable amounts of full-length product from the hybrid *Smn* gene (Fig. 3c). Supporting a heightened requirement for SMN in testis, we confirmed that there was a very high expression of SMN protein in WT testis compared to all other tissues, including brain and spinal cord (Fig. 3d). When we compared SMN levels in C/C and WT tissues, we observed a ~50% reduction in brain, liver, heart, and uterus/ovaries of C/C mice (Fig. 3e–h). However, there was no difference in SMN protein between WT and C/C testes (Fig. 3h). Although consistent with the results of MESDA, the lack of SMN reduction in testis was at odds with the drastic phenotype and may indicate one or more of the following: (i) SMN expression is reduced at an earlier developmental stage which causes defects at later time points; (ii) the cell types with low SMN expressions are already lost by P42; (iii) or the testis phenotype is an indirect result of low SMN levels in other tissues, such as the nervous system.

### C/C testis transcriptome is dramatically altered

**Figure 4:**
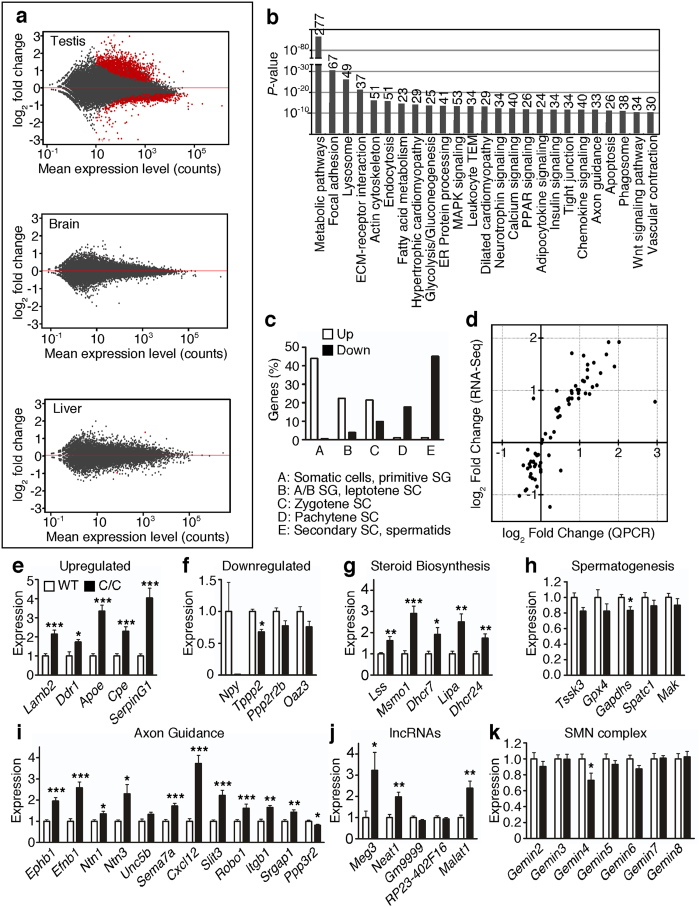
RNA-Seq reveals drastic alterations in C/C testis transcriptome. (**a**) MA plots for testis (top), brain (middle) and liver (bottom). The y-axis depicts log_2_ fold change (LFC) in gene expression in C/C compared to WT, and the x-axis depicts the mean read count for each gene between all samples. Each dot represents one gene, with red dots representing genes with significantly altered expression values (Benjamini and Hochberg (B + H) adjusted *p* value <0.05). (**b**) KEGG pathways enriched for genes with altered expression levels in C/C testis. The length of each bar represents the B + H adjusted *p* value of enrichment for each pathway. The number at the top of each bar represents the number of genes participating in each pathway with significantly altered expression. (**c**) Percentage of genes upregulated (white bars) or downregulated (black bars) in C/C testes that are classified as being primarily expressed in certain testicular cell types. Abbreviations used: spermatogonia (SG), spermatocytes (SC). (**d**) LFC in C/C testes of several candidate genes as measured by RNA-Seq (y values) and QPCR (x values). (**e,f**) QPCR showing aberrant testis expression of genes that are strongly upregulated (**e**) or downregulated (**f**). (**g–i**) QPCR for select genes involved in steroid biosynthesis (**g**), spermatogenesis (**h**) and axon guidance (**i**). (**j**) QPCR for expression of lncRNAs. (**k**) QPCR for expression of genes that encode members of the SMN complex. For (**e–k**), n = 8 mice per genotype (Statistical significance: *p < 0.05; **p < 0.01; ***p < 0.001).

We performed deep sequencing of the testis as well as brain and liver transcriptome of WT and C/C mice (Supplementary Table 2) to characterize molecular changes in these tissues (Sequence Read Archive accession number SRP062636). During initial quality control, we determined that one sample derived from a C/C testis was an outlier; it was removed from all further analysis. Using the remaining replicates, we identified 3,724 differentially expressed genes using a false discovery rate (FDR) cutoff of 0.05, with 2,186 genes upregulated and 1,538 downregulated in C/C testes (Fig. 4a). In contrast, very few genes showed aberrant expression in C/C brain and liver (Fig. 4a) Summary statistics for the 50 most significantly upregulated and downregulated genes are given in Supplementary Tables 3 and 4.

Several Kyoto Encyclopedia of Genes and Genomes (KEGG) pathways and gene ontology (GO) terms were disproportionately affected in C/C testes. The top 25 enriched pathways in C/C testis are shown in Fig. 4b, including apoptosis, cell and extracellular matrix interaction (potential problems with tissue organization and/or integrity) and cardiomyopathy and smooth muscle contraction (potential problems with blood flow and/or transport of spermatozoa to epididymis). GO term analysis was somewhat less informative (Supplementary Figs. S1–S3), although several GO terms associated with spermatogenesis were enriched in the list of downregulated genes (Supplementary Fig. S3). We also examined expression of several genes that are previously identified to be mutated in cases of oligospermia^15^; of the fourteen genes, six (*H19, Klhl10, Prm1, Shbg, Tssk4*, and *Vasa*) showed significantly altered expression in C/C testes (Supplementary Fig. 4). Aside from pathways and genes, we also detected changes suggesting altered enrichment of genes in specific groups of testicular cell types^16^. Consistent with our histological analyses, genes that are highly expressed in somatic and early spermatogenic cell types were strongly enriched, and genes expressed in late spermatocytes and spermatids were found to be downregulated (Fig. 4c). We also examined the relative expression of individual markers specific for testicular cell types (Supplementary Fig. 5). These results suggest extensive cell death of pachytene spermatocytes and/or arrest of zygotene spermatocytes in C/C testes.

We independently validated the results of RNA-Seq by quantitative PCR (QPCR) of >50 candidate genes (full list in Supplementary Table 5). There was a strong correlation (r^2^ = 0.8376) between RNA-Seq and QPCR results (Fig. 4d). Five genes in particular (*Lamb2, Ddr1, Apoe, Cpe, SerpinG1*) were highly upregulated, ranging from ~1.5–4 fold above WT (Fig. 4e). We also tested four genes, which were predicted by RNA-Seq to be highly downregulated. Of these, *Npy*, a gene that codes for neuropeptide Y pro-protein, was strongly downregulated, though *Npy* expression was highly variable. The other three genes (*Tppp2, Ppp2r2b*, and *Oaz3*) all trended downward, but the decrease was only statistically significant for *Tppp2* (Fig. 4f). Of the five steroid biosynthesis genes (*Lss, Msmo1, Dhcr7, Lipa*, and *Dhcr24*) that we tested, all were significantly upregulated (Fig. 4g). In addition, we tested 5 genes participating in spermatogenesis; however, although all trended downward, we observed a significant decrease only in *Gapdhs* (Fig. 4h). Many genes involved in axon guidance were affected; of these, the five most strongly upregulated were *Ephb1, Efnb1, Ntn3, Cxcl12*, and *Slit3* (Fig. 4i). *Ppp3r2* was the only gene in this category that was significantly downregulated (Fig. 4i). Several long non-coding RNAs (lncRNAs) had altered expression levels in C/C testes. Of the five that were validated, *Meg3, Neat1*, and *Malat1* were strongly upregulated (Fig. 4j). We examined the expression of all the *Gemins*, which code for proteins associated with SMN complex assembly and function. Only *Gemin4* was significantly downregulated (Fig. 4k).

### Apoptotic pathways are dysregulated in C/C testes

**Figure 5:**
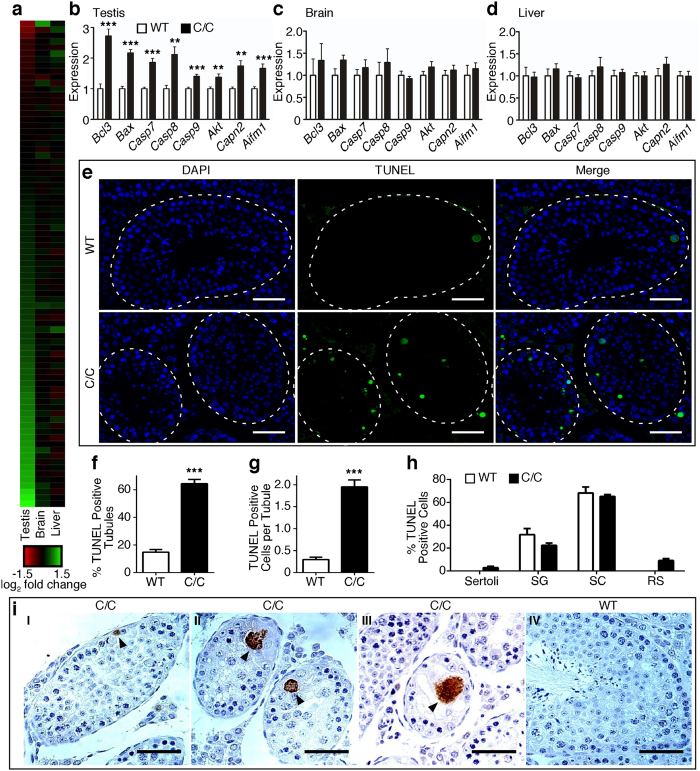
Expression of apoptosis-related genes is disproportionally affected in C/C testes. (**a**) Heat map depicting the relative expression of every gene involved in apoptosis, as annotated by the Kyoto Encyclopedia of Genes and Genomes. Each colored bar indicates the LFC of expression in C/C compared to WT. The color scale is indicated below, with red colored bars indicating genes with lower expression in C/C and green colored bars indicating genes with increased expression in C/C. (**b–d**) Relative expression of several candidate genes involved in apoptosis, as measured by QPCR in testes (**b**), brain (**c**) and liver (**d**); n = 4 mice per genotype. (**e**) Representative micrographs from TUNEL assay showing nuclei (DAPI, blue), TUNEL signal (green) and merged image. Individual seminiferous tubules are noted with dashed white outline. Scale bars represent 50 μm. (**f**) Percentage of seminiferous tubules with at least one TUNEL-positive cell. (**g**) Average number of TUNEL positive cells per seminiferous tubules. (**h**) Percentage of TUNEL-positive cells classified by cell type, including Sertoli cells, spermatogonia (SG), spermatocytes (SC) and round spermatids (RS). For (**f–h**), n = 4 mice per genotype. (**i**) Representative images of cleaved caspse 3 immunostaining. C/C testes with cleaved caspase 3-positive spermatogonia (panel I), spermatocytes (panel II) and multinucleated body (panel III) are indicated by arrowheads. WT testes showed absence of cleaved caspase 3 (panel IV). Scale bars represent 50 μm. (Statistical significance: *p < 0.05; **p < 0.01; ***p < 0.001).

Our RNA-Seq results suggested significant dysregulation of the apoptotic pathways in C/C testes (Fig. 4b), with much fewer changes in brain and liver (Fig. 5a). We confirmed by QPCR that a number of pro- (*Bax, Casp7, Casp8, Casp9, Capn2, Aifm1*) and anti- (*Bcl3, Akt*) apoptotic genes were significantly upregulated in C/C testis (Fig. 5b), but not in brain (Fig. 5c) or liver (Fig. 5d). Terminal deoxynucleotidyl transferase dUTP nick end labeling (TUNEL) staining, which detects DNA fragmentation usually associated with apoptosis, revealed a ~3-fold increase in the percentage of seminiferous tubules with at least one TUNEL-positive cell (Fig. 5e,f). We also observed a significant increase in the average number of TUNEL-positive cells per tubule (Fig. 5g). For WT testes, TUNEL-positive cells were predominantly spermatocytes and the remaining were spermatogonia (Fig. 5h). These WT TUNEL-positive cells most likely represented damaged germ cells that must be eliminated before meiotic division into abnormal spermatids^17^. Most TUNEL-positive cells in C/C testes were spermatocytes, similar to WT, but we also observed TUNEL staining of a small number of Sertoli cells and round spermatids (Fig. 5h). Since depletion of Sertoli cells can have drastic consequences for germ cell health^18^, increased apoptosis in spermatogonia and spermatocytes may be due to dysfunction of Sertoli cells, or vice versa. Immunostaining for cleaved caspase 3, a marker for apoptosis, revealed positive spermatogonia (Fig. 5i, panel I), spermatocytes (panel II) and multinucleated bodies (panel III) in C/C testes, but a lack of cleaved caspase 3-positive cells in WT testes (panel IV). This observation further underscores that apoptosis is dysregulated in C/C testes.

### *SMN2* undergoes splicing switch during testicular development

**Figure 6:**
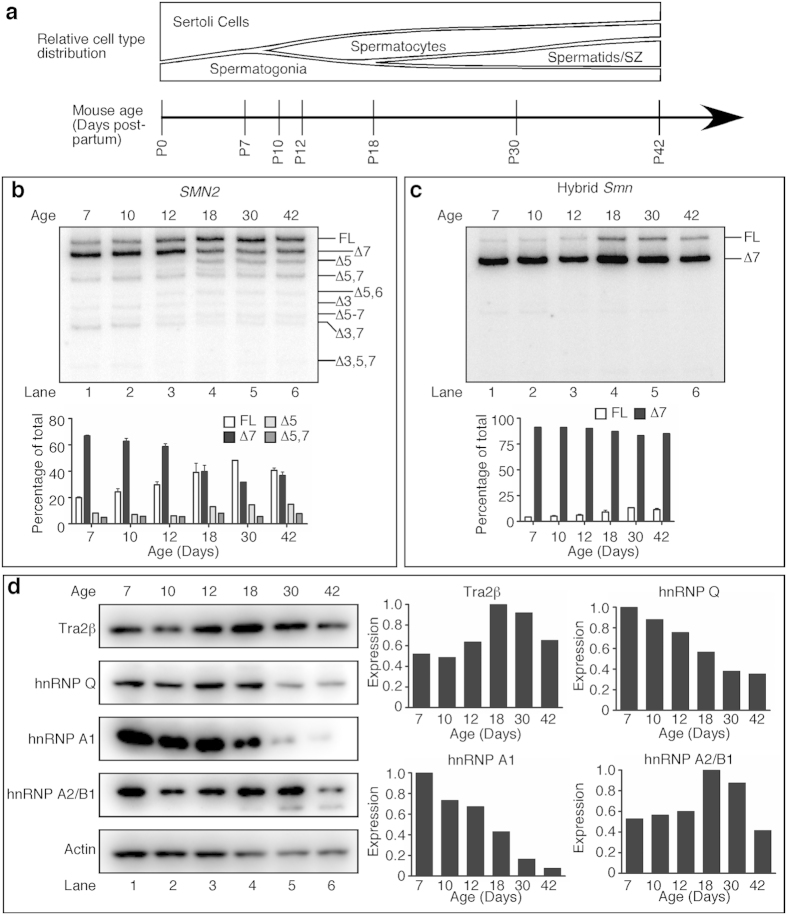
*SMN2* and hybrid *Smn* mRNA splicing changes during testis development. (**a**) Timeline of the ages and estimated WT cell type distribution of seminiferous tubules of mice used for this study. The height of each designated region represents the estimated proportion of total number of cells in an average seminiferous tubule. (**b,c**) MESDA for *SMN2* (**b**) and hybrid *Smn* (**c**); n = 2 mice per genotype. Isoform identities are given on the right side of each gel image where Δ indicates a skipped exon. Each lane is a representative example taken from two replicates. Lower panels show results of densitometric quantification of the most prominent isoforms of each gene. (**d**) Western blots for Tra2β, hnRNP Q, hnRNP A1, hnRNP A2/B1, and Actin during testis development. Equal amounts of protein from each of 3–4 C/C samples were pooled together and 15 μg of protein loaded for each time point. Densitometric quantifications are given on the right. Abbreviation: SZ, spermatozoa.

The first wave of spermatogenesis in mice occurs from P10 to P35. This process is characterized by synchronous transitions from one cell type to another and marked by predictable changes in the testis transcriptome^16^,^19^ (Fig. 6a). We employed MESDA to capture splicing of *SMN2* exons during the first wave of spermatogenesis. In particular, we compared the splicing pattern of *SMN2* in P7 testes, when spermatogonia are the predominant germ cell type, with testes undergoing meiosis (P10, P12 and P18), and near the end (P30) and after completion of the first wave of spermatogenesis (P42). We observed a dramatic shift in *SMN2* exon 7 splicing from overwhelming skipping at P7 to higher inclusion at P18 and later (Fig. 6b). We also observed increased skipping of exon 5 and decreased co-skipping of exons 3 and 7 at later time points (Fig. 6b). With regards to hybrid *Smn* (Fig. 3a), there was an increase in exon 7 inclusion starting at P18 (Fig. 6c). Our findings represent the first report of a shift in *SMN2* splicing pattern during testis development. Increased *SMN2* exon 7 inclusion coincided with the completion of initial stages of meiosis and likely occurred in spermatocytes and spermatids. *SMN2* transcripts of somatic testicular cells and spermatogonia, which are more highly represented at early time points were likely still dominated by exon 7 skipped forms, and therefore likely had a deficiency in SMN protein expression.

Previous evidence suggested that hnRNP Q and Tra2β are important for *SMN2* exon 7 inclusion in testes^9^,^10^. Thus, we examined whether changes in expression of splicing factors known to regulate *SMN2* exon 7 splicing coincided with the changes in *SMN2* splicing during testis development. hnRNP A1 and Q protein levels were highest at P7 and dropped during testis development, dramatically so in the case of hnRNP A1 (Fig. 6d). Since hnRNP Q was expressed at relatively low levels at P18 and P30, when *SMN2* exon 7 inclusion increased, it likely did not play a role in the splicing switch. hnRNP A1, however, is a known negative regulator of *SMN2* exon 7 inclusion, so its low expression in later time points is consistent with the change in exon 7 splicing. Tra2β was expressed at the highest levels at P18 and P30, when *SMN2* exon 7 inclusion is highest, followed by a decrease at P42 (Fig. 6d). hnRNP A2/B1, a negative regulator of *SMN2* exon 7 inclusion, had a nearly identical expression pattern. Overall, these data indicate that multiple splicing factors contribute to the *SMN2* splicing pattern we observe during early stages of testicular development.

**Figure 7:**
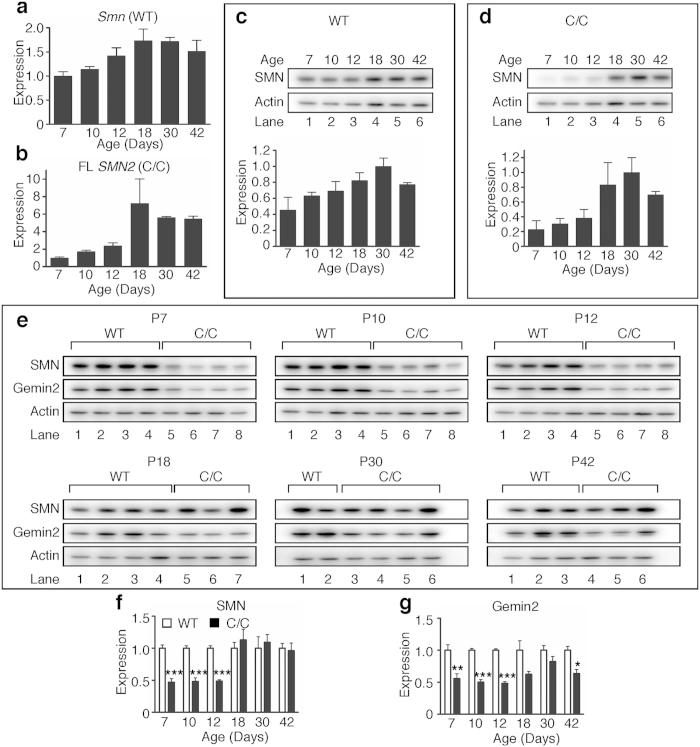
SMN mRNA and protein expression during testis development. (**a,b**) QPCR measurement of relative quantities of *Smn* transcript in WT testes (**a**) and FL (including both exons 5 and 7) *SMN2* transcript in C/C testes (**b**) during development. Expression is calculated relative to the average expression level at P7. (**c,d**) Time course of SMN protein expression in WT (**c**) and C/C (**d**) mice. Blots shown are representative examples of each time point. (**e**) SMN, Gemin2 and Actin expression in WT and C/C testes during development for each age. (**f**) Densitometric analysis for SMN western blots shown in (**e**). (**g**) Densitometric analysis for Gemin2 western blots shown in (**e**). N = 2–4 mice per genotype and age. (Statistical significance: *p < 0.05; **p < 0.01; ***p < 0.001).

In order to further verify that SMN levels are altered during testis development, we first established baseline expression during the first wave of spermatogenesis using primers targeted to mouse *Smn* mRNA in WT testes. *Smn* expression peaked at P18 and P30 and then dropped slightly at P42 (Fig. 7a). In contrast, full-length *SMN2* expression in C/C testes dramatically increased at P18 and held steady thereafter (Fig. 7b). We next examined how SMN protein expression changed during testis development. In WT testes, SMN protein (derived from *Smn*) steadily increased and peaked at P30 with a slight decrease at P42 (Fig. 7c). In C/C testes, however, SMN protein (derived from *SMN2* and hybrid *Smn*) was low until a marked increase at P18, peak expression at P30 and a subsequent decrease at P42 (Fig. 7d). When SMN protein was compared between the genotypes, C/C SMN protein level was approximately half the WT level from P7 to P12, but by P18 there was no significant difference between the genotypes (Fig. 7e,f). We also examined expression of Gemin2, a protein that interacts with SMN. Similar to SMN, Gemin2 protein level was reduced by about half from P7 to P12 in C/C testes. Gemin2 rose to near WT level by P30 followed by a drop at P42 (Fig. 7e,g). This disparity in SMN and Gemin2 levels may suggest a deficiency of SMN functions mediated by Gemin2.

### Dysregulation of alternative splicing in adult C/C testes

**Figure 8:**
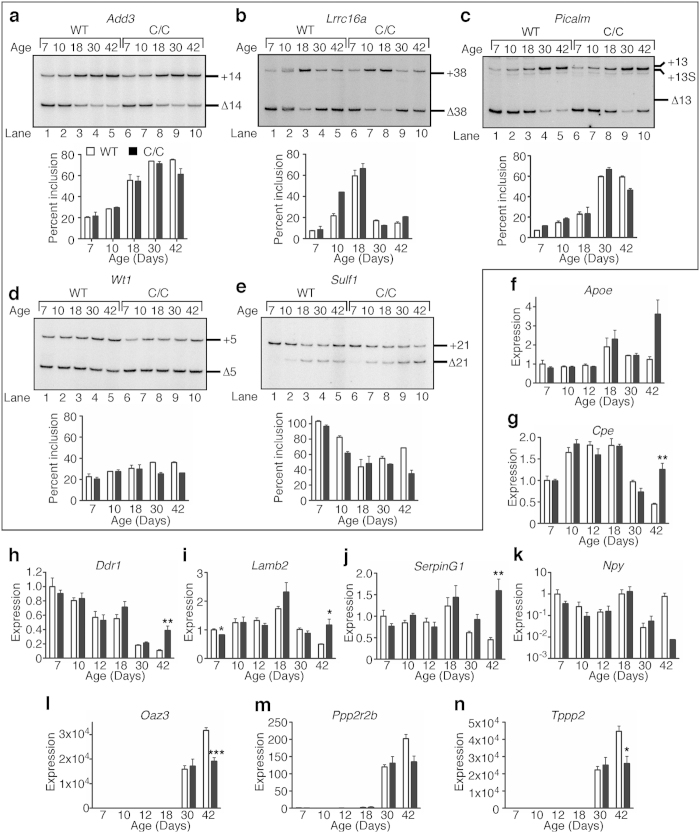
Alternative splicing and gene expression in C/C testes during spermatogenesis. Splicing patterns for *Add3* exon 14 (**a**), *Lrrc16a* exon 38 (**b**), *Picalm* exon 13 (**c**), *Wt1* exon 5 (**d**) and *Sulf1* exon 21 (**e**) during testis development. The top panels are representative autoradiograms of semi-quantitative PCR. Isoforms are labeled to the right of each gel image. The bottom panels portray autoradiogram quantification. For (**a–e**), n = 2 mice per genotype. (**f–n**) QPCR expression of candidate genes during testis development, including *Apoe* (**f**), *Cpe* (**g**), *Ddr1* (**h**), *Lamb2* (**i**), *SerpinG1* (**j**), *Npy* (**k**), *Oaz3* (**l**), *Ppp2r2b* (**m**) and *Tppp2* (**n**). *Npy* (**k**) is charted on a log_10_ scale due to large expression changes. For (**f–n**), n = 3–4 mice per genotype. (Statistical significance: *p < 0.05; **p < 0.01; ***p < 0.001).

To confirm that critical alternative splicing events are affected during C/C testes development, we examined the splicing patterns of several genes known to undergo regulated alternative splicing during spermatogenesis. *Add3* exon 14 inclusion increases during spermatogonia differentiation^11^. Both WT and C/C testes exhibited a similar increase in *Add3* exon 14 inclusion, but at P42 there was a decrease in exon 14 inclusion in C/C compared to WT (Fig. 8a). *Lrrc16a* exon 38 splicing is somewhat complicated: pattern changes from predominant exclusion to inclusion during Sertoli cell maturation and spermatogonia differentiation while round spermatids exhibit predominant exclusion^11^. In C/C testes, exon 38 inclusion followed a pattern similar to WT at P7 and P18, but not P10, and subtle differences emerged at P30 and P42 (Fig. 8b). In Sertoli cells and spermatogonia, there is a mix of *Picalm* exon 13 inclusion and exclusion, while spermatocytes and round spermatids exhibit predominant exon 13 inclusion^11^. Splicing of *Picalm* was similar for WT and C/C testes except at P42 where there was more exon 13 exclusion in C/C (Fig. 8c). Prevalence of a *Picalm* exon 13 alternative 3′ splice site was similar in WT and C/C testes (Fig. 8c).

In addition, RNA-Seq data revealed differential regulation of alternative splicing of several genes. We selected two such genes for validation. *Wt1* is a tumor suppressor gene and essential for proper urogenital development^20^. In WT testes, *Wt1* exon 5 is predominantly skipped at P7, with a gradual increase in inclusion up to P30 (Fig. 8d). C/C testes showed a similar pattern, but there was a decrease in exon 5 inclusion compared to WT testes at P30 and P42 (Fig. 8d). Skipping of *Sulf1* exon 21 is predicted to cause a frameshift eliminating the stop codon and resulting in a much longer protein. In WT testes, *Sulf1* exon 21 shifted from complete inclusion at P7 to roughly 40% at P18, then subsequently increased again (Fig. 8e). In C/C testes, exon 21 skipping remained the predominant event (Fig. 8e).

### Gene expression of candidate genes is not affected until after puberty

Having observed the preponderance of splicing changes between P30 and P42 in C/C testes, we wanted to know whether expression of key genes followed a similar pattern. We tested mRNA levels throughout development of several genes that were strongly upregulated or downregulated (Fig. 4e–f) in C/C testes by QPCR. In WT, *Apoe* was expressed highest at P18, followed by P30 and P42, and at similar levels for all other time points. However, there was a drastic increase in expression at P42 in C/C testes, although levels did not pass the threshold value we set for FDR (Fig. 8f). *Cpe* was expressed at the highest level from P10 to P18, with the lowest expression at P42. In C/C testes, expression was significantly higher than WT at P42 (Fig. 8g). In WT, *Ddr1* steadily decreased from P7 onwards. The pattern in C/C testes matched with WT until a drastic increase at P42 (Fig. 8h). *Lamb2* expression steadily increased until P18 and then fell again. In C/C, the pattern was the same with the exception that expression did not drop at P42. Expression in C/C was significantly lower at P7, although the absolute change in expression was small (Fig. 8i). *SerpinG1* expression in WT increased slightly at P18 and then dropped steadily until P42, whereas in C/C expression was highly increased at this time point (Fig. 8j).

*Npy* expression exhibited quite large variation, both between different time points and between replicates. However, the average values at each time point were quite similar between WT and C/C testes, with the exception of P42, at which C/C exhibited a striking, though not statistically significant decrease, similar to what we observed before (Fig. 8k). *Tppp2, Oaz3*, and *Ppp2r2b* exhibited strikingly similar expression patterns, with very low levels until P18, then drastically increased at P30 and P42, coinciding with the emergence of spermatozoa. For all three genes, there were no differences in expression between WT and C/C testes until P42, when expression of *Oaz3* and *Tppp2* was significantly reduced, and expression of *Ppp2r2b* trended downward (Fig. 8l–n).

Overall, none of the changes that we observed occurred until P42, after completion of the first wave of spermatogenesis. All the downregulated genes, with the exception of *Npy*, were only expressed at very low levels until P30, indicating that they are most likely spermatid- or spermatozoa-specific. This downregulation is consistent with the observation that C/C testes were generally lacking these cell types (Fig. 1). For genes upregulated in C/C testes, expression in WT was generally lowest at P42, except *Apoe*. Interestingly, expression of *Apoe* in C/C testes at P42 was higher than WT at any time point, indicating that there is a mechanism beyond developmental dysregulation causing increased expression of this gene.

## Discussion

In this report, we employed the C/C mouse to evaluate for the first time the role of SMN in mammalian sex organ development and fertility. We observed severe impairment of male reproductive organ development characterized by smaller testes, widespread degeneration of seminiferous tubules, loss of post-meiotic cells, and a drastic reduction in male fertility in C/C mice. These surprising findings underscore a requirement for a relatively high level of SMN for the development and maintenance of male reproductive organs in mammals. A report published in 1986 implicated testis dysfunction in two mild SMA patients^21^, although genetic diagnoses were not available at that time. A subsequent report briefly mentioned unexplained clinical symptoms due to the influence of male sex in type 3 SMA patients^22^. Although the authors did not elaborate on the exact nature of the symptoms, they conveyed the necessity for an animal model of mild SMA to examine male specific pathology. Hence, our finding of a unique testicular phenotype in C/C mice fills a critical knowledge gap with respect to SMN function.

The testis has unique RNA metabolism, including transcription, splicing and 3′-end processing^11^,^23^,^24^. Given the prominent role of SMN in RNA metabolism and the presence of Gemin3, an SMN-interacting protein, in germ cell-specific chromatoid bodies^25^,^26^,^27^, it is not unexpected that SMN plays a critical role in testis development and spermatogenesis. However, it was somewhat unexpected that we did not observe drastically altered gene expression until an adult age, when SMN expression in C/C testes reached high levels due to high inclusion of *SMN2* exon 7. Gemin2 has a stabilizing effect on SMN and its interaction with SMN is critical for most SMN functions^28^. Low level of Gemin2 in C/C testes at P42 is likely to impact all SMN functions dependent on SMN-Gemin2 interactions. A dramatic change in the transcriptome of C/C testis at P42 underscores perturbations in pathways critical to spermatogenesis and testis function, including steroid biosynthesis, apoptosis, and spermatogenesis itself (Fig. 4). There was an enrichment of markers of interstitial cells and somatic cells of the seminiferous tubules and general loss of expression of genes enriched in post-meiotic cell types (Fig. 4, Supplementary Fig. 4). Intergenic lncRNAs and transposons expressed during meiosis are degraded through the Piwi-interacting RNA (piRNA) pathway in which Tudor domain-containing proteins play an essential role^29^. SMN being a tudor domain-containing protein and given the propensity of tudor domains to forge interactions among themselves^26^,^30^, it is possible that SMN directly or indirectly affects piRNA pathway.

It is known that lncRNAs are differentially regulated in testis, especially during spermatogenesis^31^,^32^. The most highly affected lncRNAs in C/C testes were *Meg3, Malat1* and *Neat1. Meg3* is expressed from paternally imprinted loci in mammals^33^ and is known to act as a tumor suppressor^34^. *Malat1* functions by affecting the phosphorylation of splicing factors^35^, and *Neat1* serves as a scaffold for the formation of paraspeckles in which many RNA binding proteins are sequestered^36^. All types of non-coding RNAs and untranslated regions impact the availability of RNA binding proteins and control the fate of mRNAs during spermatogenesis^37^. Altered expression of lncRNAs reveals a novel function of SMN in controlling the levels of freely available RNA-binding proteins in testes.

Apoptosis plays an essential role in spermatogenesis as it eliminates damaged meiotic and post-meiotic cells in testes^17^,^38^. SMN has anti-apoptotic properties^39^,^40^,^41^ and interacts with Bcl-2 to protect cells from Fas-mediated apoptosis^42^,^43^. Very low levels of SMN trigger DNA fragmentation in muscles and motor neurons of SMA patients^44^,^45^. Our finding of testicular defects and increase in TUNEL-positive cells in C/C seminiferous tubules suggests a necessity for much higher levels of SMN to prevent the dysregulation of apoptotic pathways in this tissue. Sertoli cells regulate germ cell survival through the Fas system; Fas ligand decorating the surface of Sertoli cells interacts with Fas present on germ cells^46^. Sertoli cells are the major cell type in testes until P18^19^, the time during which SMN is most strongly reduced in C/C testes. Since SMN modulates Fas-mediated apoptosis, low SMN in Sertoli cells could disrupt the normal regulation of this process. Further, low SMN causes defects in snRNP assembly and expression levels^25^, resulting in accumulation of the aberrantly spliced mRNA isoforms, a condition to which Sertoli cells are particularly sensitive^46^,^47^. SMN has also been implicated in the resolution of double-stranded breaks requiring expression of functional H2AX^48^. Hence, increased TUNEL staining in C/C testes could be due to an increase in the unresolved double-stranded breaks arising from meiotic recombination.

Substantial downregulation of mRNA encoding neuropeptide Y proprotein suggests possible denervation of C/C testes and is likely a downstream effect of the low levels of SMN at the early stages of testicular development. Supporting this argument, SMN-independent defects leading to mild SMA display abnormal male reproductive organ development and defective spermatogenesis. The most notable among these is the mutations in *Vps54* gene leading to a motor neuron disease similar to mild SMA and defects in male reproductive organ development^49^. Plastin 3 coded by *PLS3* is a protective modifier of SMA caused by the loss of *SMN1*^50^. Interestingly, the protective effect of Plastin 3 is not fully penetrant and appears to be specific to females. A recent study in rat testis implicates the role of Plastin 3 in spermatogenesis^51^. We did not observe aberrant expression of *Vps54* or *Pls3* genes in C/C testis, excluding a direct role of these genes in testicular defects in C/C mice. However, our results combined with the above findings suggest an overlapping pathway of neurodegeneration and male reproductive organ development.

About 50% of male infertility cases, affecting >5% of the population worldwide, are caused by genetic abnormalities^15^,^52^. More than 20% of these cases represent nonobstructive azoospermia (NOA) in which semen contains little or no sperm^15^,^53^. A genome-wide association study (GWAS) reported in 2012 linked only 6 genes to male fertility traits^54^, none of which were linked to pre-mRNA splicing, which is uniquely regulated during spermatogenesis. However, a GWAS conducted on Chinese men focused on serine-arginine (SR) protein-coding genes and found a strong correlation between NOA and mutations in *SFRS9*, encoding SR protein SRp30c^55^. Incidentally, SRp30c regulates SMN levels by promoting inclusion of exon 7 during pre-mRNA splicing^56^. Other studies provide additional evidence supporting the role of SMN in spermatogenesis. For example, the transcription factor SP1 is predicted to modulate SMN levels in both mouse and humans^57^. A critical role for SP1 in mouse male germ cell development has been previously described^58^. Further, a recent study suggested that PRMT5, which plays an essential role in assembly of spliceosomal snRNPs along with SMN^27^, is required for germ cell survival during spermatogenesis in mice^59^. Finally, upregulation of SMN upon activation of phosphatidylinositol 3-kinase (PI3K)/AKT and its positive impact on disease phenotype of severe SMA has been demonstrated^60^. Abrogation of PI3K/AKT pathway has been shown to affect spermatogenesis but not oogenesis^61^. Our finding of a reproductive phenotype and loss of fertility in C/C males, but not females, are consistent with those observations. Incidentally, several human studies have revealed fewer females than males afflicted with the milder forms of SMA^62^,^63^,^64^. Given these findings, it is rather expected that SMN plays a critical role in testis development and male germ cell survival in mammals.

Humans are unique in harboring *SMN2*, a gene whose function is still unknown. Generally, *SMN2* is considered to be dispensable for survival, since enough SMN could be generated from *SMN1*. Our findings provide a strong rationale that the preservation of *SMN2* during evolutions of humans was driven by the heightened requirement of SMN in male reproductive organ development. A very specific splicing switch of *SMN2* to elevate SMN levels in adult testis in humans appears to be part of a fine regulatory mechanism that remains to be further understood. The presence of *SMN2* in SMA patients carries profound significance due to its ability to serve as a promising therapeutic target. Several pre-clinical studies employing *SMN2*-containing severe mouse models of SMA have shown extraordinary survival benefits^3^. However, gender-specific efficacy of drugs and reproductive phenotype of severe SMA mice that survive much longer upon treatment, remain unknown. Lack of gender-specific data in pre-clinical studies in neurodegenerative diseases has been cited as one of the major sources of misinterpretations and faulty conclusions^65^. Our findings validate those concerns and provide a repertoire of histological and molecular markers that could be employed for the careful monitoring of male reproductive organ health as an essential component of drug development process for SMA.

## Materials and Methods

All methods were performed in accordance with the approved biosafety and radiation safety guidelines of Iowa State University (ISU), adhering to the federal and state guidelines. All animal experiments were carried out in accordance with the approved protocols by the Institutional Animal Care and Use Committee (IACUC) of ISU, adhering to the guidelines of American Veterinary Medical Association (AVMA), United States Health and Human Services (US HHS), United States Department of Agriculture (USDA) and State of Iowa.

### Mice

Mice heterozygous for the C allele^12^ (*Smn*^*C*/+^) on the C57BL/6 background were purchased from Jackson Laboratory (stock number 008714). Breeding cages with one male and one female heterozygote were set up and maintained at ISU to generate the mice used for all experiments. This cross generated WT mice (*Smn*^+/+^) and C/C mice (*Smn*^*C/C*^). Pups were genotyped and sacrificed at the appropriate ages. Unless otherwise stated, all experiments were carried out at P42.

### Fertility assessment

Monogamous breeding pairs were established between three- to five-month-old mice and followed for ninety days. Once established, females were checked every twelve hours for a vaginal plug for the first five days. The number of litters and number of pups per litter were recorded. After ninety days mice were weighed, euthanized and tissues were collected. Testis mass and epididymal sperm count were determined for male mice.

### Epididymal sperm counting

P60 WT and C/C mice were anesthetized with isoflurane and killed by cervical dislocation. The epididymides were dissected, minced with a fresh number 10 disposable scalpel and placed in 3 ml Dulbecco’s Modified Eagles’ Medium supplemented with 10% fetal bovine serum (Life Technologies) for 15 minutes at 37 °C. After incubation, the cell suspension was triturated 15 times. Dilutions of the sperm were prepared in water and incubated at room temperature for two minutes. The diluted cell suspension was loaded on a haemocytometer, allowed to settle, and only sperm with a head and tail were counted.

### Tissue collection

For RNA and protein isolation, P7, P10, P12, P18 and P30 WT and C/C mice were deeply anesthetized with isoflurane and decapitated. Tissues were removed and stored at −80 °C for future use. P42 mice were deeply anesthetized with 100 mg/kg ketamine and 10 mg/kg xylazine, the carotid artery was pierced and blood collected and the mouse was subsequently transcardially perfused with phosphate buffered saline (PBS; 137 mM NaCl, 2.7 mM KCl, pH 7.4). Unless otherwise stated, tissues were removed, flash frozen on dry ice, and stored at −80 °C for future use. Blood was allowed to clot at room temperature for 15 minutes, centrifuged at 2,000 × *g* for 10 minutes at 4 °C and the serum stored at −80 °C for future use.

For histology, P42 WT and C/C mice were weighed, deeply anesthetized with 100 mg/kg ketamine and 10 mg/kg xylazine and transcardially perfused with PBS followed by 4% paraformaldehyde (pH 7.4). After perfusion, the sex organs were removed, weighed with an analytical balance (Ohaus) and post-fixed overnight in modified Davidson’s fixative (Electron Microscopy Diatome) at 4 °C. Organs were processed, embedded in paraffin, sectioned and stained with hematoxylin and eosin (H&E).

### Histological analysis of tissue

H&E stained sections were analyzed by a board-certified Veterinary Pathologist to determine any pathological changes. Photographs of sections were taken with a Nikon microscope with SPOT Advanced software (Diagnostic Imaging, Inc.).

We utilized a previously published 10-point scoring system to assess the progression of spermatogenesis within the seminiferous tubules^13^. Twenty seminiferous tubules from all areas of each testis were scored. The pathologist was blinded as to genotype of each sample. The scoring system is as follows:

1 - No cells in tubular cross section.

2 - Sertoli cells only.

3 - Only spermatogonia present.

4 - No spermatozoa, no spermatids, but fewer than 5 spermatocytes present.

5 - No spermatozoa, no spermatids, but many spermatocytes present.

6 - No spermatozoa, but fewer than 5-10 spermatids present.

7 - No spermatozoa, but many spermatids present.

8 - All stages of spermatogenesis present, but fewer than 5–10 spermatozoa.

9 - Many spermatozoa present, but germinal epithelium disorganized with marked sloughing or obliteration of lumen.

10 - Complete spermatogenesis.

### Terminal deoxynucleotidyl transferase dUTP nick end labeling (TUNEL) staining

Three-μm formalin-fixed paraffin-embedded testis cross sections from P42 mice were subjected to TUNEL staining with the *In situ* Cell Death Detection Assay, Fluorescein (Roche) following the manufacturer’s protocol. For the negative control, two slides were incubated with only Labeling Solution (no enzyme). For the positive control, one slide was incubated with 2 U/μl DNase I, recombinant (Roche) for 10 minutes at room temperature to induce DNA fragmentation and then incubated as per the kit protocol. After labeling, sections were coverslipped with VectaShield Mounting Medium with DAPI (Vectastain) and imaged. Eight to ten micrographs from all regions of the testis were captured for analysis. TUNEL positive bodies were counted in at least 50 tubules from all regions of the testis for quantification.

### Immunohistochemistry

Three-μm formalin-fixed paraffin-embedded testis cross sections from P42 mice were deparaffinized in xylene and rehydrated to distilled water. The sections were subjected to antigen retrieval for 30 minutes at 95 °C in antigen retrieval buffer (10 mM sodium citrate, 0.05% Tween-20, pH 6.0). The sections were then incubated in 0.3% hydrogen peroxide (Fisher) for 30 minutes to block endogenous peroxidase activity. Immunostaining was performed using the VECTASTAIN Elite ABC kit (Vector Laboratories) following the manufacturer’s protocol. The primary antibody, rabbit anti-cleaved caspase 3 (Cell Signaling; 9661) was diluted 1:100 and incubated with sections for 90 minutes at room temperature. Detection was performed using the ImmPACT DAB substrate (Vector Laboratories). Slides were subsequently stained with modified Mayer’s hematoxylin (Fisher), dehydrated in an ethanol gradient, coverslipped with Permount (Fisher) and imaged.

### Testosterone enzyme linked immunosorbent assay (ELISA)

Total serum testosterone was measured using the Mouse/Rat Testosterone ELISA kit (CalBiotech) using the manufacturer’s instructions. A standard curve was constructed using GraphPad Prism v6.0 using a sigmoidal 4-parameter logistics fit and testosterone concentration (ng/ml) were determined based on this curve.

### Protein isolation

Tissue was homogenized in ten volumes radioimmunoprecipitation assay (RIPA) buffer (Boston Biotechnologies) with 1X HALT protease inhibitor (Thermo Fisher Scientific). The tissue was sonicated on ice three times for 10 seconds each with one minute between pulses at speed 2 with a Microson Ultrasonic Cell Disruptor (Misonix). The homogenate was incubated on ice for 30 minutes, centrifuged at 12,000 rpm for 15 minutes at 4 °C and the supernatant transferred to a new tube. If the supernatant still appeared cloudy after centrifugation, the previous step was repeated. Protein concentration was determined with the BioRad Protein Assay. For immature (P7-P30) testes, tissue was homogenized in a glass tissue grinder (Wheaton), then processed the same as above, omitting the sonication step.

### Immunoblotting, silver staining, and densitometric analysis

Protein lysates were diluted with 2X Laemmli sample buffer (BioRad) with 10% β-mercaptoethanol (Sigma-Aldrich). Samples were boiled for 10 minutes and then centrifuged at maximum speed for 5 minutes at room temperature. Different amounts of protein were loaded depending on the tissues: 12.5–15 μg for testis, 30 μg for uterus/ovaries and brain, 50 μg for liver and 75 μg for heart. For silver staining, protein was separated on 10% SDS-PAGE gels and then stained with the BioRad Silver Stain Plus kit according to the manufacturer’s protocol. For western blotting, protein was separated on 10% SDS-PAGE gels and then transferred to Immobilon polyvinylidene fluoride (PVDF) membrane (Millipore) using the Turbo Transfer system (BioRad). Membranes were briefly washed in 1X Tris-buffered saline with Tween 20 (TBST; 50 mM Tris, 150 mM NaCl, 0.05% Tween-20, pH 7.4), and then incubated in 5% nonfat milk prepared in TBST. Membranes were then incubated in the appropriate primary antibody diluted in 5% milk: mouse anti-SMN (BD Biosciences; 610646) diluted 1:2,000 (for heart, liver, and uterus/ovaries) or 1:5,000 (for testes), mouse anti-Gemin2 (Clone 2E17; Sigma G6669) diluted 1:500; mouse anti-hnRNPA1 (Clone 9H19; Abcam; ab5832) diluted 1:10,000; mouse anti-hnRNPA2/B1 (Abcam; ab6102) diluted 1:1,000; mouse anti-hnRNP Q (Sigma; R6353) diluted 1:800; goat anti-Tra2β (Abcam; ab31353) diluted 1:1,000; or rabbit anti-Actin (Sigma; A2066) diluted 1:2,000. Incubations were performed overnight at 4 °C with shaking, except anti-Actin, which was incubated for 1 hour at room temperature. Blots were then washed three times in TBST for at least 10 minutes with shaking and incubated in the appropriate secondary antibody: goat anti-mouse IgG, goat anti-rabbit IgG or donkey anti-goat IgG all conjugated to horseradish peroxidase (HRP) for 1 hour at room temperature or for 30 minutes at 37˚C with shaking. Blots were then developed with either Clarity HRP substrate (BioRad) or West Femto substrate (Thermo Scientific Pierce) and visualized with the BioSpectrum AC Imaging System (UVP). For each blot, the mean intensity of each band was determined using ImageJ software. For each sample, the mean intensity of the protein of interest was divided by the mean intensity of Actin. For direct comparisons between WT and C/C, the WT average was set at 1.0 and all other samples were expressed relative to this value. For all time-course experiments within a genotype, the maximum expression was set at 1.0 and all other samples were expressed relative to this value.

### Deep sequencing and analysis of C/C transcriptome

RNA was isolated from P42 testes, liver, and brain (n = 4 WT, n = 3 C/C for testis, n = 2 males, 2 females per genotype for liver and brain) using Trizol reagent according to the manufacturer’s instructions. The isolated RNA was quantified using a UV spectrophotometer and 10 μg of total RNA was subjected to digestion with RQ1 RNase-free DNase (Promega). After DNase treatment, the RNA was repurified using phenol:chloroform extraction followed by ethanol precipitation. Quality and quantity of samples was assessed on an Agilent bioanalyzer using an RNA Nano chip (Agilent). 300 ng of RNA was used as input for poly(A) + RNA enrichment using the NEBNext poly(A) mRNA Magnetic Isolation Module (New England Biolabs) and RNA-Seq libraries were generated using the NEBNext Ultra Directional RNA Library Prep Kit (New England Biolabs). During library preparation, barcode sequences were introduced for identifying sequences from each biological replicate and 6–7 libraries were pooled and run per lane on an Illumina HiSeq 2500 using a 100-cycle single-end program.

After sequencing, contaminating adapter sequences and low-quality ends were removed using the cutadapt program^66^. After trimming, reads were mapped to the mouse genome (version GRm38/mm10) using Tophat^67^. For transcript identification, the Gencode annotation was used (http://www.gencodegenes.org/). In order to identify differentially regulated transcripts, read counts were obtained for all Gencode annotated genes using HTSeq-count^68^ and altered expression levels were tested using the DESeq package implemented in R^69^. GO term and KEGG pathway analysis were performed using the WebGESTALT web server (http://bioinfo.vanderbilt.edu/webgestalt/). Candidate alternatively spliced exons were identified using MATS^70^.

### Quantitative PCR and semi-quantitative RT-PCR

RNA was isolated from testes using the same protocol as total RNA extraction used for deep sequencing, except 30 μg of RNA was used for DNase treatment and cleanup was performed on RNeasy columns (Qiagen). 2.5 μg of total RNA was converted to cDNA using SuperScript III reverse transcriptase (Life Technologies) using random primers (Promega). For quantitative PCR, 1.5 μl of a 1:20 dilution of cDNA (equivalent to cDNA produced from 9.375 ng of RNA) was used as template in a 20 μl reaction containing 300 nM forward and reverse primers and 1X FastStart Universal SYBR Green Master containing ROX reference dye (Roche). QPCR was performed using a Stratagene MX3005P thermocycler (Agilent) and relative expression levels were determined using the ΔΔCt method using β-Actin as a reference gene. For comparisons in adult tissues, values are expressed as relative to WT average. For time-course experiments, values are expressed as relative to WT average at P7. MESDA was performed as described previously^14^. For other semi-quantitative PCR reactions, 0.5 μl of cDNA was used per 20 μl PCR reaction using Taq polymerase (New England Biolabs) in the presence of 0.8–1.2 μCi of [α-^32^P] dATP. 6 μl of PCR product was separated on a 5% (for MESDA) or 8% (for other semi-quantitative PCR) TBE polyacrylamide gel, dried, and exposed to a phosphorimager screen. Gel images were scanned using a Fujifilm FLA-5100 and densitometric quantification was carried out using Multi Gauge software (Fujifilm). For all reactions labeled with [α-^32^P] dATP, values were corrected by dividing by the number of A/T base pairs in the product. All primer sequences appear in Supplementary Table 6.

### Statistical analysis

Statistical analysis was performed with GraphPad software. All data are presented as mean ± standard error of the mean (S.E.M.) unless otherwise stated. Data on sex organ weights, sperm count, fertility, TUNEL staining, and protein densitometry were analyzed with two-tailed unpaired Student’s *t* test. QPCR results were analyzed with unpaired two-tailed unpaired Student’s *t* test with multiple hypothesis correction using the Benjamimi and Hochberg method. Seminiferous tubule score means were analyzed with the Mann-Whitney U test. For all statistical analyses, *p* < 0.05 was considered significant.

## Additional Information

**How to cite this article**: Ottesen, E. W. *et al*. Severe impairment of male reproductive organ development in a low SMN expressing mouse model of spinal muscular atrophy. *Sci. Rep.* **6**, 20193; doi: 10.1038/srep20193 (2016).

## Supplementary Material

Supplementary Information
