# Supplementary material for: Severe impairment of male reproductive organ development in a low SMN expressing mouse model of spinal muscular atrophy
Source: Sci Rep. 2016 Feb 2;6:20193. doi: 10.1038/srep20193 (PMC4735745; doi:10.1038/srep20193)
Supplement: Supplementary Information [file srep20193-s1.pdf]

Severe impairment of male reproductive organ development in a low SMN expressing mouse model of spinal muscular atrophy

Supplementary Figures and Tables

Eric W. Ottesen, Matthew D. Howell, Natalia N. Singh, Joonbae Seo, Elizabeth M. Whitley, and Ravindra N. Singh

Supplementary Figure Legends

Supplementary Fig. 1. GO terms enriched in all significantly altered genes. The length of each bar represents the B+H adjusted *p* value of enrichment for each pathway. GO terms are characterized as representing biological processes (a), molecular functions (b), or cellular components (c)

Supplementary Fig. 2. GO terms enriched in significantly upregulated genes. The length of each bar represents the B+H adjusted *p* value of enrichment for each pathway. GO terms are characterized as representing biological processes (a), molecular functions (b), or cellular components (c)

Supplementary Fig. 3. GO terms enriched in significantly downregulated genes. The length of each bar represents the B+H adjusted *p* value of enrichment for each pathway. GO terms are characterized as representing biological processes (a) or cellular components (b). No GO terms corresponding to specific molecular functions were significantly enriched.

Supplementary Fig. 4. Expression of markers of particular cell types. mRNA expression level of cell-type markers in C/C testes as measured by RNA-Seq. Value is expressed as expression relative to WT. (Statistical significance: *$p<0.5$; **$p<0.01$; ***$p<0.001$)

Supplementary Fig. 5. Expression of genes mutated in oligospermia. mRNA expression level in allele C testes of genes whose mutation causes oligospermia, as measured by RNA-Seq. Value is expressed as expression relative to WT. (Statistical significance: *$p<0.5$; **$p<0.01$; ***$p<0.001$)

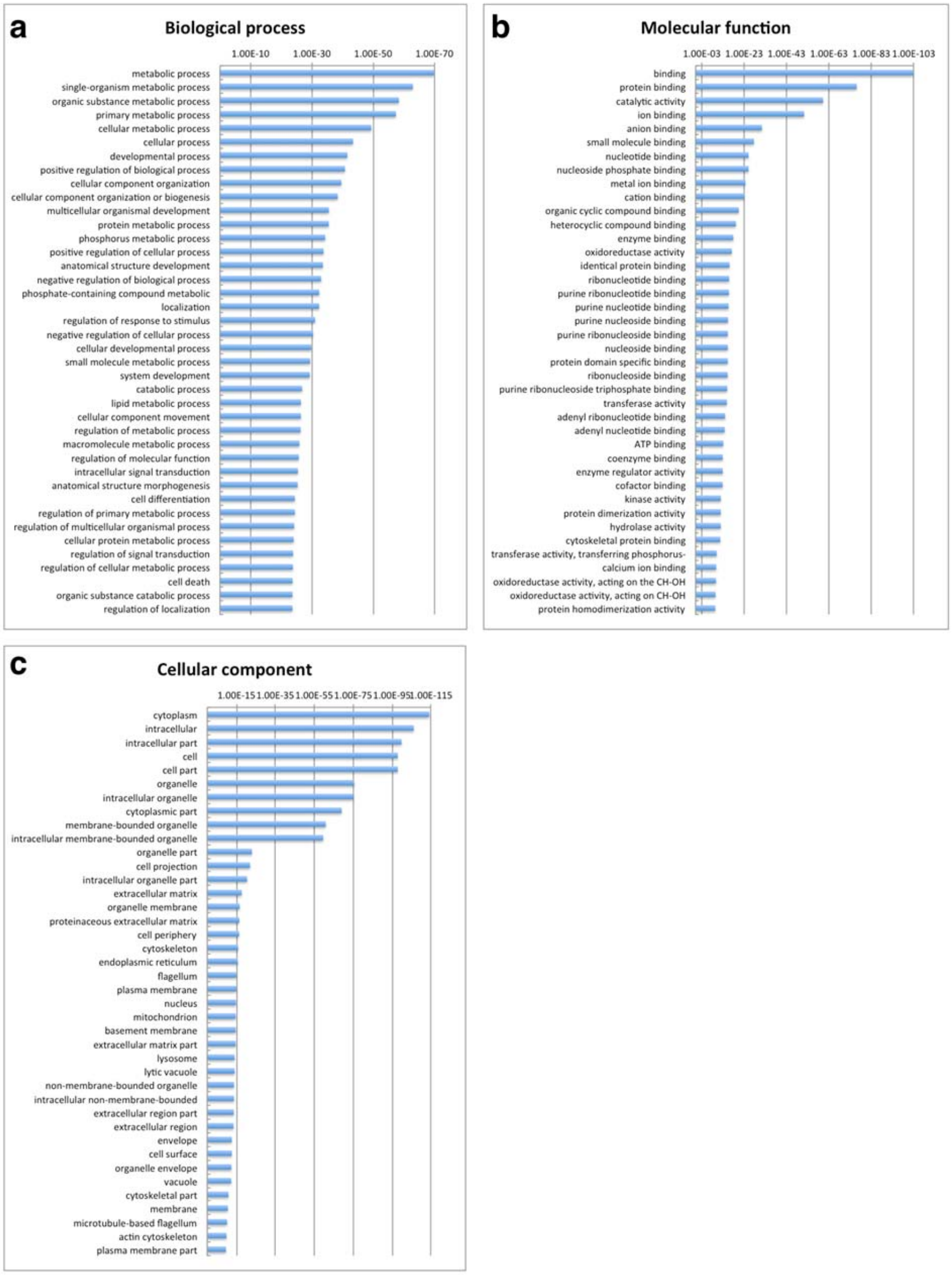

Supplementary Figure 1

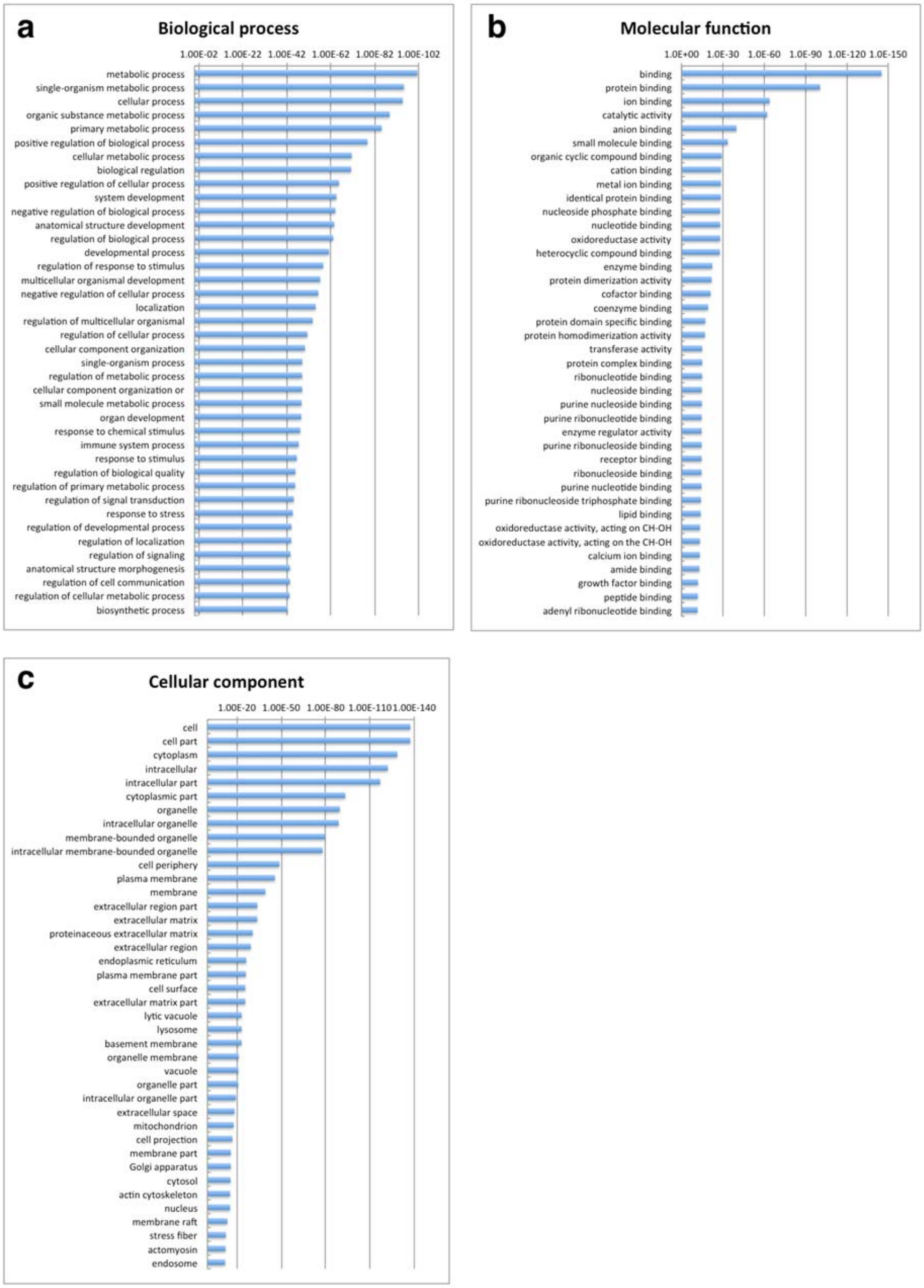

Supplementary Figure 2

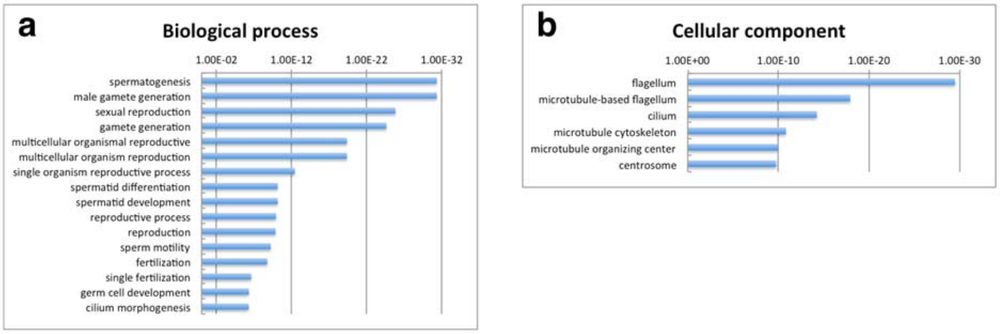

Supplementary Figure 3

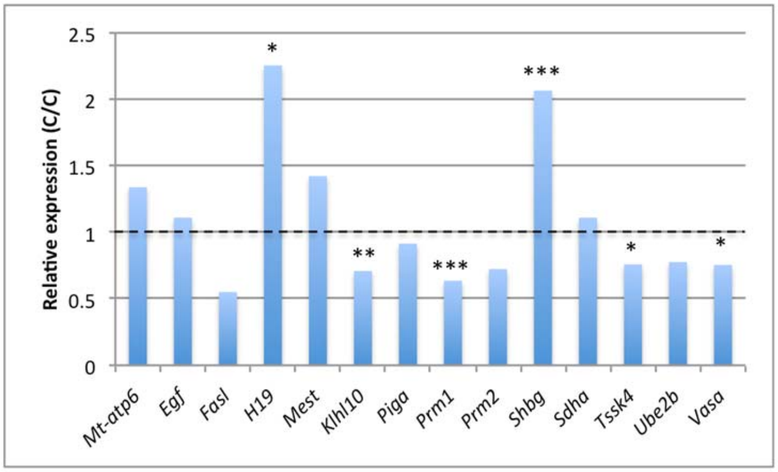

Supplementary Figure 4

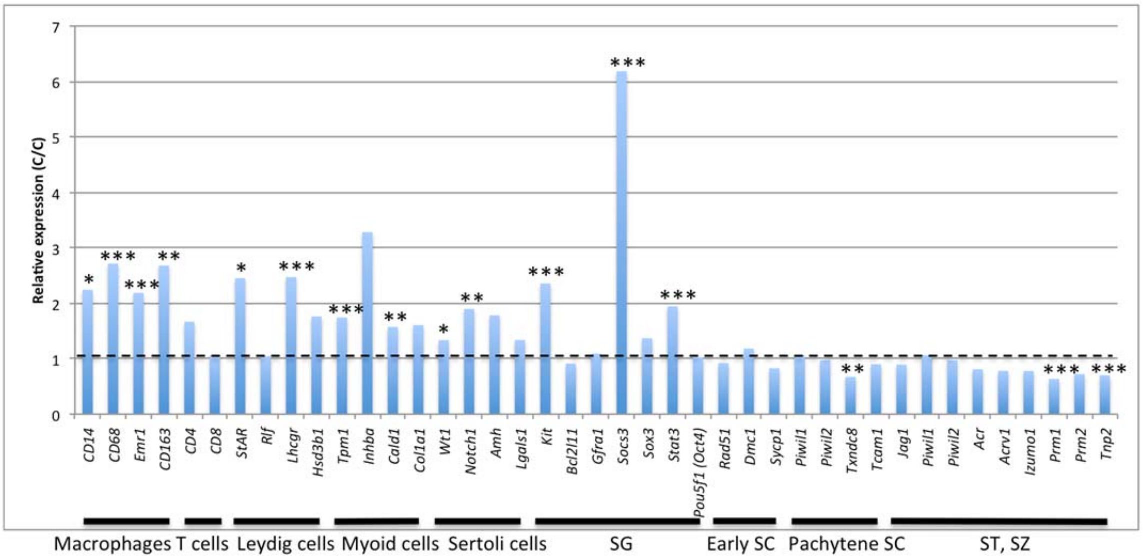

Supplementary Figure 5

Supplementary Table 1: Details on Male Mice used for Fertility Study					
**Male**	**Male *Smn* Genotype**	**Female *Smn* Genotype**	**Total Pups Sired in 90 days (Total Litters)**	**Relative Testis Mass***	**Sperm Count (x10^6)**
**1**	WT	C/C	11 (3)	6.4	9.15
**2**	WT	C/C	15 (2)	6.31	16.5
**3**	WT	C/C	13 (2)	5.62	20
**4**	C/C	WT	3 (1)	1.73	0.165
**5**	C/C	WT	0 (0)	1.5	1.59
**6**	C/C	WT	0 (0)	1.72	0
**7**	C/C	WT	0 (0)	1.62	0.99
**8**	C/C	WT	0 (0)	1.14	1.05
**9**	C/C	WT	0 (0)	1.43	1.71
**10**	C/C	C/C	8 (1)	2.25	2.4
**11**	C/C	C/C	0 (0)	2.02	3.96

*Relative testis mass determined by dividing the testis mass by total body weight.

Supplementary Table 2: Sequencing and mapping of WT and C/C Transcriptome							
Organ	Sex	Mouse Identifier	Genotype	Barcode	Millions of reads	% Mapped	% Multiply mapped
Testis	M	817	WT	ACAGTG	30.54	89.56%	6.34%
	M	841	WT	ATCACG	26.66	87.67%	6.59%
	M	1218	WT	GCCAAT	32.05	86.60%	6.75%
	M	1222	WT	CGATGT	23.75	86.29%	6.81%
	M	818*	C/C	GATCAG	20.92	84.22%	7.70%
	M	839	C/C	ACAGTG	23.49	91.80%	5.88%
	M	1395	C/C	TAGCTT	30.25	88.66%	5.87%
Liver	M	841	WT	ATCACG	25.29	79.89%	9.70%
	M	1218	WT	GATCAG	28.85	79.86%	12.43%
	F	826	WT	CGATGT	25.29	80.48%	11.40%
	F	1241	WT	GGCTAC	20.85	81.26%	9.98%
	M	818	C/C	ACAGTG	26.70	79.54%	9.88%
	M	839	C/C	ATCACG	27.08	81.18%	11.70%
	F	827	C/C	GCCAAT	28.78	80.02%	12.95%
	F	1097	C/C	CGATGT	25.27	80.78%	10.03%
Brain	M	817	WT	ATCACG	37.43	86.74%	6.65%
	M	841	WT	GATCAG	32.32	88.05%	6.78%
	F	1241	WT	CGATGT	31.57	86.85%	6.94%
	F	1244	WT	TAGCTT	36.17	92.56%	7.06%
	M	1395	C/C	ACAGTG	19.12	88.65%	6.98%
	M	818	C/C	ATCACG	31.98	94.70%	6.25%
	F	827	C/C	GCCAAT	27.11	87.40%	7.25%
	F	833	C/C	CGATGT	29.09	95.34%	6.48%

* - Outlier sample, which was removed from downstream analyses

Supplementary Table 3: Most highly upregulated genes identified by RNA-Seq				
Gene symbol	Ensembl ID	Log2 Fold Change	Standard error	Adjusted P value
Hist1h1c	ENSMUSG00000036181	2.03	0.20	8.71E-21
Lamb2	ENSMUSG00000052911	1.67	0.17	1.67E-19
Ddr1	ENSMUSG00000003534	1.71	0.17	1.67E-19
Apoe	ENSMUSG00000002985	1.92	0.19	2.26E-19
Cpe	ENSMUSG00000037852	1.39	0.14	1.42E-18
Serping1	ENSMUSG00000023224	1.93	0.20	1.42E-18
Aldh1a1	ENSMUSG00000053279	1.47	0.16	1.14E-17
Sparcl1	ENSMUSG00000029309	1.70	0.18	2.28E-17
Aebp1	ENSMUSG00000020473	1.82	0.20	8.94E-16
Timp2	ENSMUSG00000017466	1.12	0.13	8.45E-15
Wnt6	ENSMUSG00000033227	1.55	0.18	1.63E-14
Igfbp3	ENSMUSG00000020427	2.45	0.29	1.63E-14
Rcn3	ENSMUSG00000019539	1.97	0.23	2.14E-14
Lamb1	ENSMUSG00000002900	1.43	0.17	3.76E-14
C1ra	ENSMUSG00000055172	2.02	0.25	2.07E-13
Arpc1b	ENSMUSG00000029622	1.22	0.15	2.36E-13
Rgs11	ENSMUSG00000024186	2.16	0.26	2.43E-13
Clec3b	ENSMUSG00000025784	2.05	0.26	8.20E-13
Ildr2	ENSMUSG00000040612	2.63	0.33	9.31E-13
Pdgfra	ENSMUSG00000029231	1.55	0.19	1.24E-12
Prss35	ENSMUSG00000033491	1.96	0.25	1.64E-12
Kit	ENSMUSG00000005672	1.24	0.16	2.78E-12
Col6a6	ENSMUSG00000043719	2.38	0.30	3.29E-12
Lrp1	ENSMUSG00000040249	1.39	0.18	6.16E-12
Lgmn	ENSMUSG00000021190	1.11	0.14	1.00E-11
Ndrg2	ENSMUSG00000004558	1.42	0.19	1.00E-11
Dcn	ENSMUSG00000019929	1.87	0.24	1.02E-11
Tspan4	ENSMUSG00000025511	1.70	0.22	1.27E-11
Lipg	ENSMUSG00000053846	1.29	0.17	1.29E-11
Phlda3	ENSMUSG00000041801	2.44	0.32	1.34E-11
Mpeg1	ENSMUSG00000046805	1.53	0.20	1.41E-11
9030617O03Rik	ENSMUSG00000021185	2.73	0.36	1.59E-11
Sulf2	ENSMUSG00000006800	1.29	0.17	2.82E-11
Aox1	ENSMUSG00000063558	1.53	0.20	3.04E-11
Kif1c	ENSMUSG00000020821	1.20	0.16	3.26E-11
Tshz2	ENSMUSG00000047907	1.26	0.17	3.69E-11
Ctsz	ENSMUSG00000016256	1.77	0.24	4.75E-11

Supplementary Table 3: Most highly upregulated genes identified by RNA-Seq cont.				
Gene symbol	Ensembl ID	Log2 Fold Change	Standard error	Adjusted P value
Meg3	ENSMUSG00000021268	1.69	0.23	5.23E-11
Osr2	ENSMUSG00000022330	1.73	0.23	5.23E-11
Lamc3	ENSMUSG00000026840	1.34	0.18	5.65E-11
Myh9	ENSMUSG00000022443	1.18	0.16	6.22E-11
Vim	ENSMUSG00000026728	1.08	0.15	7.12E-11
Aplp1	ENSMUSG00000006651	1.39	0.19	9.74E-11
C1qa	ENSMUSG00000036887	1.89	0.26	9.81E-11
Timp1	ENSMUSG00000001131	3.08	0.42	1.12E-10
Aldh2	ENSMUSG00000029455	1.24	0.17	1.13E-10
Cyp27a1	ENSMUSG00000026170	1.56	0.21	1.13E-10
Akr1cl	ENSMUSG00000025955	1.58	0.22	1.15E-10
Ccdc8	ENSMUSG00000041117	1.45	0.20	1.51E-10
Cdkn1a	ENSMUSG00000023067	1.43	0.20	1.53E-10

Supplementary Table 4: Most highly downregulated genes identified by RNA-Seq				
Gene symbol	Ensembl ID	Log2 fold change	Standard error	Adjusted P
Tmem56	ENSMUSG00000028132	-1.06	0.13	1.58E-12
Tmsb15a	ENSMUSG00000060726	-1.23	0.17	6.05E-11
Hypm	ENSMUSG00000040456	-1.03	0.14	1.43E-10
Tppp2	ENSMUSG00000008813	-1.02	0.14	3.04E-10
Oaz3	ENSMUSG00000028141	-0.89	0.13	3.82E-10
Hist1h1t	ENSMUSG00000036211	-1.55	0.22	6.97E-10
1700012A03Rik	ENSMUSG00000029766	-1.00	0.15	2.07E-09
1700027A15Rik	ENSMUSG00000101968	-0.95	0.15	2.50E-08
Ppp2r2b	ENSMUSG00000024500	-0.92	0.15	3.62E-08
1700009N14Rik	ENSMUSG00000028287	-0.83	0.13	3.95E-08
1700031F10Rik	ENSMUSG00000047046	-1.31	0.21	7.50E-08
1700015G11Rik	ENSMUSG00000094445	-0.81	0.13	8.62E-08
Spata18	ENSMUSG00000029155	-0.84	0.14	1.00E-07
Lrrc8b	ENSMUSG00000070639	-0.72	0.12	2.07E-07
Abcb5	ENSMUSG00000072791	-3.26	0.54	2.23E-07
1700016H13Rik	ENSMUSG00000029320	-0.75	0.13	4.34E-07
Yam1	ENSMUSG00000098178	-1.04	0.18	5.05E-07
Hist1h2ba	ENSMUSG00000050799	-1.06	0.18	5.44E-07
Clmn	ENSMUSG00000021097	-0.74	0.13	8.70E-07
Gm23935	ENSMUSG00000076258	-0.95	0.17	1.14E-06
Pdzd8	ENSMUSG00000074746	-0.72	0.13	1.37E-06
Nsun4	ENSMUSG00000028706	-0.79	0.14	1.71E-06
Tnp1	ENSMUSG00000026182	-0.62	0.11	1.84E-06
H2afb1	ENSMUSG00000062651	-0.72	0.13	2.16E-06
Lars2	ENSMUSG00000035202	-0.92	0.17	2.52E-06
Npy	ENSMUSG00000029819	-3.29	0.60	2.67E-06
1700020G17Rik	ENSMUSG00000101801	-0.86	0.16	2.79E-06
Gm11780	ENSMUSG00000085155	-0.83	0.15	2.98E-06
Klk1b8	ENSMUSG00000063089	-0.87	0.16	3.37E-06
Prom1	ENSMUSG00000029086	-0.68	0.13	3.79E-06
Prkar2a	ENSMUSG00000032601	-0.72	0.13	3.79E-06
Cdrt4	ENSMUSG00000042200	-0.73	0.14	4.09E-06
Gm498	ENSMUSG00000031085	-0.77	0.14	4.16E-06
BB014433	ENSMUSG00000049008	-0.71	0.13	4.17E-06
Rpl39l	ENSMUSG00000039209	-0.68	0.13	4.24E-06
1700019O17Rik	ENSMUSG00000036574	-0.69	0.13	4.35E-06
Lrrc74a	ENSMUSG00000059114	-0.75	0.14	4.51E-06

Supplementary Table 4: Most highly downregulated genes identified by RNA-Seq cont.				
Gene symbol	Ensembl ID	Log2 fold change	Standard error	Adjusted P
Gm5893	ENSMUSG00000011350	-0.66	0.12	5.20E-06
1700001C19Rik	ENSMUSG00000047150	-0.71	0.13	5.26E-06
Dbil5	ENSMUSG00000038057	-0.79	0.15	5.66E-06
St6galnac2	ENSMUSG00000057286	-0.76	0.14	5.66E-06
Spem1	ENSMUSG00000041165	-0.77	0.14	6.10E-06
Bpifa3	ENSMUSG00000027482	-0.80	0.15	7.41E-06
Lrrc57	ENSMUSG00000027286	-0.74	0.14	7.52E-06
1700080E11Rik	ENSMUSG00000032566	-0.84	0.16	8.56E-06
Akap4	ENSMUSG00000050089	-0.72	0.14	9.03E-06
Ubqlnl	ENSMUSG00000051437	-0.68	0.13	9.06E-06
Mir6236	ENSMUSG00000098973	-1.07	0.21	9.06E-06
Acsl1	ENSMUSG00000018796	-0.71	0.14	9.07E-06
1700001K19Rik	ENSMUSG00000056508	-0.74	0.14	9.60E-06

Supplementary Table 5: QPCR validation of RNA-Seq

Gene	Selected GO terms/KEGG pathways	Allele C relative expression	
		QPCR	RNA-Seq
Unc5b	Netrin receptor, apoptotic process, axon guidance	1.327	1.992
Ntn3	Receptor binding, axonogenesis	2.292	2.371
Gpx4	Response to oxidative stress, spermatogenesis	0.824	0.727
Casp8	Apoptotic process, cysteine-type endopeptidase activity	2.112	2.277
Bcl3	DNA damage response, negative regulation of apoptotis	2.721	2.141
Spatc1	Gamma-tubulin binding, centrosome	0.823	0.635
Cdkl2	Cyclin-dependent protein serine/threonine kinase activity	0.995	0.697
Hdac7	B cell activation, histone deacetylation, vasculogenesis	1.964	1.798
Bax	Sertoli cell proliferation, apoptotic process	2.171	2.254
Ntn1	Axon guidance, proteinaceous extracellular matrix	1.352	1.529
EphB1	Axon guidance, angiogenesis, cell adhesion	1.956	1.927
HoxD10	Regulation of transcription, embryonic limb morphogenesis	2.304	2.813
Mak	Cyclin-dependent protein serine/threonine kinase activity	0.892	0.730
Tssk3	Protein serine/threonine kinase activity, spermatogenesis	0.900	0.612
Gapdhs	Glycolytic process, sperm motility	0.834	0.659
Sema7a	Axon guidance, integrin binding	1.725	1.987
Itgb1	Axon guidance, cell-matrix adhesion	1.619	1.778
Npy	GPCR binding, neuropeptide signaling pathway	0.012	0.103
Lamb2	Integrin binding, axon guidance, basement membrane	2.145	3.174
Lss	Cholesterol biosyntheic process	1.628	1.752
Meg3	DNA methylation, genetic imprinting	3.225	3.224
Cxcl12	Chemokine activity, germ cell development, axon guidance	3.723	2.747
Ddr1	Collagen binding, protein tyrosine kinase activity	1.737	3.274
Msmo1	Cholesterol biosyntheic process	2.906	2.517
Neat1	Protein binding, nuclear body organization	1.977	2.009
Slit3	Axon guidance, cell differentiation	2.217	1.922
Tppp2	Tubulin binding	0.679	0.493
Apoe	Cholesterol homeostatis, lipid homeostasis	3.352	3.795
Dhcr7	Cholesterol biosynthetic process	1.920	2.015
Gm9999	Unknown function	0.855	0.624
Ppp3r2	Calcium-dependent protein SR phosphatase activity	0.813	0.752
Oaz3	Polyamine biosynthetic process	0.773	0.538

Supplementary Table 5: QPCR validation of RNA-Seq continued

Gene	Selected GO terms/KEGG pathways	Allele C relative expression QPCR	RNA-Seq
Lipa	Cholesterol biosynthetic process	2.511	2.092
RP23-402F16	Unknown function	0.931	0.558
Fus	RNA binding	1.356	1.400
Robo1	Axon guidance	1.653	1.667
Srgap1	Axon guidance, Rho protein signal transduction	1.425	0.690
Ppp2r2b	Apoptotic process, signal transduction	0.757	0.528
Cpe	Peptidase activity, insulin processing	2.298	2.628
Serping1	Negative regulation of complement activation	4.043	3.798
Dhcr24	Cholesterol biosynthetic process	1.740	1.928
Malat1	Regulation of alternative mRNA splicing	2.385	1.906
Efnb1	Axon guidance, cell differentiation	2.576	2.889
Srpk1	Protein serine/threonine kinase activity, RNA splicing	0.955	0.852
Lsm2	RNA splicing	0.874	0.773
Rbmxl2	RNA splicing	0.790	0.910
Rbmx	RNA splicing, DNA-templated transcription	1.310	1.558
SFPQ	RNA splicing, DNA repair, DNA-templated transcription	1.186	1.068
Gemin2	Spliceosomal snRNP assembly	0.903	0.863
Gemin3	Helicase activity, Spliceosomal snRNP assembly	0.996	0.678
Gemin4	Spliceosomal snRNP assembly	0.729	0.739
Gemin5	snRNA binding, spliceosomal snRNP assembly	0.929	0.843
Gemin6	Spliceosomal snRNP assembly	0.873	0.777
Gemin7	Spliceosomal snRNP assembly	1.008	0.994
Gemin8	Spliceosomal snRNP assembly	1.025	1.037
Ptbp1	RNA splicing	1.082	1.124
Ptbp2	RNA splicing	1.152	0.845
Hnrnpa1	RNA splicing	1.264	1.152

Supplementary Table 6: Primers used for QPCR		
Gene	Forward Primer	Reverse Primer
Gemin2	GGAGAGTGCAGATCGAAGCA	GCCACTTGTTGTTGTTGCCA
Gemin3	AGTCGGGCACTGGAAAAACT	ACCAGGAGATCCAACAGCAA
Gemin4	GGAAGACCTCAACACGACGT	GCAGGGAAGGCAGTGAGAAT
Gemin5	ACAGCATAACCCCTGGAAGC	AGCCAGGACTTTGCCATCTC
Gemin6	GCAACTGGTAACCATGAGCG	GGCCCATGATTCCAGTCACA
Gemin7	ATCCCCCAGAGTCACAGGAA	AGGTCTGTGGCTCCAAACTG
Gemin8	AGAGGGAATGAAGCACGGTG	GGTGGTTGAAGTACAGGCCA
Ptbp1	AGTCTGGAAACCTGGCCTTG	GATCTTCAGGACGGTGCCAA
Ptbp2	GGCAACAGAGGAAGCAGCTA	GAACTACTTGCGCACGTTGG
Hnrnpa1	ACGAGAGTCTGAGGAGCCAT	TCCACTTCTTCCACAGTGGC
Srpk1	ACAAAGCCCAAAGGAAGCCT	TCCCCAGCCCAATTTTCGAA
Lsm2	GGCAAGGATGTAGTCGTGGA	AGGGTATTTCTCAGGGTCTGTG
Rbmxl2	TCTTCATCGGCGGTCTCAAC	TTCTCGAAGGTGACGAAGGC
Rbmx	GTTCGTAGCAGCAGTGGACT	TCCATCATCTCTCGGGGACA
Sfpq	GCCAGCAGCAAGAAAAGCAT	TGTGCCATGCTGAGCAAAAC
HisH1C	GGCATCCTGGTGCAAACCAA	CCTTTTTGGGTGTGGCAGCA
Unc5b	ACGGCCAACTACACCTGTGT	ACAGAAGGCGCCTCCATTGA
Netrin 1	TGTCTCAACTGCCGCCACAA	TTGCAGGGACATTGGCCAGT
Netrin 3	ATGCCGCTTCAACATGGAGC	TGGCAGTCACAAGCTCTGCA
Casp7	AACCGTCCACAATGACTGCTCT	TGTCACGCCATCTTTCCCGTAA
Casp8	AGCTGCGGGATCCAGACAAT	TGCCAGCATGGTCCTCTTCT
Casp9	GGACCGTGACAAACTTGAGCAC	ATCTCCATCAAAGCCGTGACCA
Akt1	ACACCTTTATCATCCGCTGCCT	CTCTTCAGCCCCTGAGTTGTCA
Apaf1	AGAGATCCACACAGGCCATCAC	ATCACACCGTGAACCCAACTCA
Capn2	TTGACGCCAATGAGGAGGACAT	CCACTCCCATCTTCATCCAGCA
Aifm1	CCGTTCGGAGAGTGAGACAGAG	TAGCACAATCCCCACCACAACT
Bcl3	ACAGCGGCCTCAAGAACTGT	TGGCACTTTGGTCTGGGGAT
Bax	CCCACCAGCTCTGAACAGATCA	TGTCCACGTCAGCAATCATCCT
Fus	AGTTGAAGGGTGAGGCAACAGT	TTCCACCACCCCGATTGAAGTC
HoxD10	CGAAGTGCAGGAGAAGGAAAGC	AGCGTTTGGTGCTTGGTGTAAG
EphB1	GCAGCAGGAAACGAGCTTACAG	CGGCTTCATTGGGGTCCTCATA
Hdac7	CTCTTGAGCCCTTGGACACAGA	GGGTTTGTAGCGCAACTTCAGG
Spatc1	ACGCCCCACCGAAAGTCTAA	TGGCACAGGTCCTCATCCAA
Cdkl2	AGTCTCCCAGTCTGGCGTTG	CGTCACCGACCAGCAGTTCT
Mak	CGAGGCCATCCAGCTCATGA	CCCGGCAGGCTGATCAAGTA
Tssk3	CTTTGCCAAGGTGCTACCCAAG	ATACAGGACCACACCCATGCTC
H1fnt	GCTAAGGACCTAGTGCGTTCCA	GTACTGCTGCTCTTTGGCACTG
Gpx4	CGCTCCATGCACGAATTCTCAG	GGCCAGGATTCGTAAACCACAC

Supplementary Table 6: Primers used for QPCR continued		
Gene	Forward Primer	Reverse Primer
Hist1h1a	TCTACTGCCACGGAGAAACCTG	CCACTGCGCTCTTTGGAAGAAG
Hils1	AAATGGTGGCTGGAGACCAAGA	TTATGTACCCGCTTGTCAGCCA
Dnahc8	AGGCTGAGCTGGATAAAGTGCA	GGTAGGAGAGAAAGCCTGTGCA
Gapdhs	GTGTCAGTTGTGGACCTGACCT	GGGATTGCCGTTAAAGTCCGTG
Efnb1	AACGTCCAATGGGAGCTTGGAG	TAGTCAACTGCTCGGGTGTCAC
Sema7a	CGGAAGCTCTATGTGACCTCCC	GTGTGGCTCCGCTGGATTAATG
Cxcl12	GGTAAACCAGTCAGCCTGAGCT	TTGTTGTTCTTCAGCCGTGCAA
Slit3	GTGGCGACTGACAAGGACAATG	CCACACTGTGAAACTGGCCATC
Itgb1	GTGAGACATGTCAGACCTGCCT	TCCTTGCAATGGGTCACAGGAT
Robo1	CTCCCCCACATAGCAACAGTGA	GTAGGACACAGCAGCTGGAGAA
Ppp3r2	GCTGGTGGACAAGAGCATCTT	AAGTCTTCTTGACCGTGTTCTACA
Ppp3cc	AGGAAGAACCAAGCAACTGGCT	TGAAGTTTGGGAGCCAGTAGGG
Srgap1	CTGTACTTCCGAGGGCTGGAAA	CGTCACTGTACTGGGAGAGGTG
Npy	CTGACCCTCGCTCTATCTCTGC	CAGTGTCTCAGGGCTGGATCTC
Tmsb15a	ACCAGACTTGTCGGAAGTGGAG	ACTCCTTCTCTTGCTCGATGGT
Tppp2	GGCCAAGAATGCCAGAACCATC	TGGTGTCTGTCAACCGGTCTAC
Oaz3	GGATCAAGGCAACCGAGAAAGC	TCTAACCACCTCGAAGCCCATG
Ppp2r2b	ACCGGAAGATCCAAGCAACAGA	AACTTGCTGCGGAGGTAGTCAT
Lamb2	GGGTGAGAGACAGAAGGCAGAG	ACCGGCAGAGTTCAGAGACTTC
Ddr1	GACTCCTCTGACACCTTCCCAC	TGAGAAGCAGCAGCAGGATGAT
Apoe	GTCACATTGCTGACAGGATGCC	CTTCCTGGACCTGGTCAGACAG
Cpe	TCTCTGTGGACGGGATAGACCA	CAAGCTCAAAGTCCACCCCAAC
Serping1	CCACTTACCTGACGATGCCTCA	TGGCAGACACCTGAAGATCTGG
Lss	AGGAGCACGTTTCTCGGATCAA	AGGGCAGAAACTCAGGTCTGTG
Msmo1	GCTGTGCAGTCATTGAGGACAC	GGGATGTGCGTATTCTGCTTCG
Dhcr7	GCCTGGACCCTCATTAACCTGT	AAGTGGTCATGGCAGATGTCGA
Lipa	AATTTTGCTTCAGGCCCGCTAC	GCGCAAAGCTCCTTCATGATGA
Dhcr24	GATGGACATCCTGGAGGTGGAC	GGTCATCAAGCTCAGGCAACAC
Meg3	CCATCTCCACAGAAGAGCAGCT	CCCACGCAGGATTCCAGATGAT
Neat1	TGTTCACCATGGCAAGCAGATG	ACAAGCTGACTTCAACGATGGC
Gm9999	GGGACCCCTCTGGAAGTAAACA	TGTGCGATTACTTTATGCGAAACCT
RP23-402F16.1	ATGAAGACTTCTCCACCCAGCC	CCACCCTACCTACTGTAGTACGC
Malat1	TTCATGGAGCTGCTCAGGACTT	ACCTGAAGTCAAGACACCTGCA
Smn	AGAACAGAACACTCAGGAGAATGA	CTTTCCTGGTCCTAATCCTGA
FL SMN2	TTCCTTCTGGACCACCAATAA	TCTATGCCAGCATTTCTCCTTAATTTAAG

Supplementary Table 7: Primers used for semiquantitative PCR		
Gene	Forward Primer	Reverse Primer
Add3	CTAACCCATTCAGCCACCTC	CATCATGCATCTCGTCCTTG
Lrrc16a	CTTCCACCCATCCTCAAAAA	CTCCCTTGGTGACCATCTTT
Picalm	GCCAAATGACCTGCTTGACT	CCAGAATCTACAGCAACATTCG
Wt1	CCAGCTTGAATGCATGACCTGG	CTCACTCTCATACCCTGTGCCG
Sulf1	CACACAGTAGAACGGAGCATCT	ACTAACCTTCCCATCCATCCCA
SMN2	CGCGGGTTTGCTATGGCGAT	TTAGTGCTGCTCTATGCCAGCATTT
Hybrid Smn	GAAGAATGCCACAACTCCCTTGA	TTAGTGCTGCTCTATGCCAGCATTT
